# CX3CL1 deficiency ameliorates acute kidney injury by inhibiting macrophage mitochondrial dysfunction and mtDNA-cGAS-STING signaling

**DOI:** 10.1038/s41420-025-02915-w

**Published:** 2025-12-13

**Authors:** Qiming Gong, Fahui Liu, Yuqing Huang, Dehui Li, Tingting Zhou, Chen Zeng, Yan Jiang, Huang Wei, Yong Xu

**Affiliations:** 1 Department of Endocrinology and Metabolism, The Affiliated Hospital of Southwest Medical University, Luzhou, Sichuan 646000 China; 2 Department of Nephrology, Affiliated Hospital of Youjiang Medicial University for Nationalities, Baise, 533000 China; 3 Metabolic Vascular Diseases Key Laboratory of Sichuan Province, Luzhou, 646000 China; 4 Sichuan Clinical Research Center for Nephropathy, Luzhou, 646000 China; 5 Sichuan-Chongqing Joint Key Laboratory of Metabolic Vascular Diseases, Luzhou, Sichuan 646000 China; 6 Key Laboratory of Medical Research Basic Guarantee for Immune-Related Diseases Research of Guangxi, Baise, 533000 China; 7 Xiamen Cell Therapy Research Center, the First Affiliated Hospital of Xiamen University, School of Medicine, Xiamen University, Xiamen, China

**Keywords:** Biomarkers, Diseases

## Abstract

Dysregulated mitochondrial dynamics and macrophage-driven inflammation are essential contributors to the pathogenesis of acute kidney injury (AKI). Although the chemokine CX3CL1 has been associated with inflammatory responses, its role in AKI, particularly in regulating macrophage polarization and mitochondrial function, remains unclear. In this study, we investigated the therapeutic potential of CX3CL1 inhibition in a lipopolysaccharide (LPS)-induced AKI model. Our results found that CX3CL1 deficiency could significantly ameliorate renal dysfunction and attenuate inflammatory responses. RNA sequencing revealed that CX3CL1 deficiency alters macrophage subpopulations and gene expression profiles in the kidney, particularly affecting pathways related to immune responses and mitochondrial function. Mechanistically, the absence of CX3CL1 promotes macrophage polarization from a pro-inflammatory M1 phenotype toward an anti-inflammatory M2 phenotype. Furthermore, CX3CL1 inhibition improves mitochondrial dynamics, alleviates mitochondrial dysfunction, and reduces oxidative stress and mitochondrial DNA (mtDNA) leakage, thereby preserving mitochondrial integrity. Notably, CX3CL1 knockdown suppresses activation of the cGAS-STING pathway, a key mediator of inflammation triggered by cytosolic mtDNA. We also observed that these effects appear to be mediated through stabilization of mitochondrial transcription factor A (TFAM). Collectively, these findings identify CX3CL1 as an essential regulator of macrophage mitochondrial function and inflammation in AKI, offering a potential therapeutic target for mitigating kidney injury.

## Introduction

Sepsis-induced acute kidney injury (AKI) is a life-threatening condition characterized by a systemic inflammatory response and is associated with high morbidity and mortality, particularly in intensive care settings, where its incidence frequently exceeds 50% [1]. Despite its prevalence and clinical severity, no specific therapeutic interventions have been established for AKI, highlighting the urgent need for novel treatment strategies [2]. Among the various approaches under investigation, immune modulation has shown promise, especially regarding the central role played by kidney-resident macrophages in the initiation and progression of AKI [3]. Macrophages can be classified into classically activated (M1) and alternatively activated (M2) subsets, representing pro-inflammatory and anti-inflammatory phenotypes, respectively, and the dynamic balance between them is important for regulating tissue inflammation, injury, and subsequent repair processes [4]. Although promoting M2-type polarization is considered a potential therapeutic avenue for treating AKI, the molecular mechanisms governing macrophage phenotypic transitions during AKI remain incompletely understood.

Mitochondrial dysfunction has been increasingly recognized as a key contributor to the pathogenesis of AKI, with several underlying mechanisms including impaired mitochondrial biogenesis, accumulation of mitochondrial DNA (mtDNA) mutations, elevated oxidative stress, and disruptions in mitochondrial dynamics [5, 6]. Inflammation-induced mitochondrial dysfunction further aggravates oxidative stress and contributes to renal cellular injury, thereby accelerating AKI progression [7, 8]. Recent evidence indicates that mitochondrial damage can amplify inflammatory responses through the release of mtDNA into the cytosol, which subsequently activates the cGAS-STING signaling pathway, an important regulator of inflammation and tissue damage in AKI [9]. Despite these insights, the pathways that trigger mitochondrial dysfunction and drive macrophage polarization in AKI remain inadequately characterized.

CX3CL1, the only member of the CX3C chemokine family, is highly expressed in kidney macrophages [10]. It has been reported that inhibition of CX3CL1 confers protective effects against renal inflammation [11], and its expression is significantly upregulated in AKI, where it is associated with increased oxidative stress and apoptosis in renal tissues [12]. In addition, CX3CL1 inhibition has shown therapeutic benefits in various kidney disorders, including diabetic nephropathy, lupus nephritis, and renal fibrosis [13–15]. However, the mechanistic relationship between CX3CL1, mitochondrial dysfunction, and macrophage-mediated inflammation in AKI remains poorly understood.

This study showed that CX3CL1 is strongly upregulated in LPS-stimulated macrophages and during AKI. In an LPS-induced AKI model, CX3CL1-knockout mice exhibited significantly reduced kidney injury, which was associated with preservation of mitochondrial function and structural integrity, as well as suppression of mitochondrial ROS generation and mtDNA leakage in renal macrophages. Mechanistically, CX3CL1 knockdown in both AKI mice and RAW264.7 cells promoted macrophage polarization toward an anti-inflammatory M2 phenotype and alleviated mitochondrial dysfunction. Collectively, our findings indicate that CX3CL1 plays a pivotal role in linking mitochondrial dysfunction with macrophage-driven inflammation in AKI.

## Methods and materials

### Reagents and antibodies

Lipopolysaccharide (LPS; L2880) was purchased from Sigma-Aldrich (St. Louis, MO, USA). Antibodies against CX3CL1 (ab25088), NGAL (ab23477), Kim-1 (ab316854), CD206 (ab64693), iNOS (ab178945), and MTCO1 (ab14705) were obtained from Abcam (Cambridge, USA). Antibodies against CD86 (sc-19617), IL-10 (sc-8438), TNFα (sc-52746), Tomm20 (sc-17764), dsDNA (sc-58749), TFAM (sc-166965), and Bax (sc-20067) were purchased from Santa Cruz Biotechnology (CA, USA). Antibodies targeting cGAS (DF12574), STING (DF12574), phosphorylated-TBK1 (p-TBK1, AF8190), total TBK1 (DF7026), phosphorylated-IRF3 (p-IRF3, AF2439), total IRF3 (DF6895), ATPB (DF12111), ATP5A (DF3806), SOD2 (AF5144), PGC-1α (AF5395), Mfn2 (DF8106), DRP1 (DF7037), and Fis1 (DF12005) were purchased from Affinity Biosciences (OH, USA). Antibodies against NDUFB8 (12794-1-AP), SDHB (10620-1-AP), and UQCRC2 (14742-1-AP) were purchased from Proteintech (Wuhan, China). TFAM-targeting siRNA and CX3CR1-targeting siRNA were bought from Santa Cruz Biotechnology (CA, USA). Recombinant CX3CL1/Fractalkine Chemokine protein was purchased from PeproTech (400-26, Rocky Hill, NJ).

### Experimental animals

C57BL/6 mice were purchased from Shanghai Genechem Animal Co., Ltd. and housed in a specific pathogen-free (SPF) facility at Youjiang Medical University for Nationalities (No. SYXK 2022-0004). The animals were maintained under controlled conditions with a 12-h light/dark cycle, a temperature of 20–24 °C, 50% relative humidity, and provided with sterile water and food ad libitum. All animal experiments were approved by the Institutional Review Board of the Ministry of Health of the People’s Republic of China and the Ethics Committee of Youjiang Medical University for Nationalities (Approval no. 2021103001).

### Establishment of the CX3CL1-knockout and LPS-induced AKI mouse model

The CX3CL1-knockout (KO) mouse model was established as described previously [16]. Briefly, the male mice (aged 8–10 weeks) were randomly assigned to three groups (*n* = 5). AKI was induced by intraperitoneal injection of LPS (10 mg/kg, diluted in 0.9% saline) for 24 h. Kidney and blood samples were collected at the time of euthanasia for subsequent analyses.

### Assessment of renal function by serum biochemical analysis

Serum creatinine (Scr) and blood urea nitrogen (BUN) levels were measured using commercial kits from Nanjing Jianjian Bioengineering Institute, following the manufacturer’s protocols (catalog numbers: C013-2-1 and C011-2-1, respectively).

### Transcriptome sequencing and bioinformatic analysis of kidney tissues

Total RNA was extracted from mouse kidney tissues using TRIzol reagent (Thermo Fisher, 15596018). mRNA was enriched using Dynabeads Oligo(dT) magnetic beads (Thermo Fisher, 25-61005) and fragmented with the NEBNext Magnesium RNA Fragmentation Module (NEB, E6150S) at 94 °C. First-strand cDNA synthesis was performed using SuperScript II (Invitrogen, 1896649), followed by second-strand synthesis using E. coli DNA polymerase I (NEB, M0209) and RNase H (NEB, M0297). The second strand was labeled with dUTP (Thermo Fisher, R0133) to construct strand-specific libraries. Libraries were sequenced using paired-end 150-bp reads (PE150) on the Illumina NovaSeq 6000 platform. Raw reads were filtered for quality and aligned to the reference genome to quantify gene expression levels. RNA-Seq data can be found in the supplemental material.

Macrophage-related gene expression among treatment groups was visualized using the IOBR package. Differential gene expression analysis was performed using the Kruskal-Wallis non-parametric test with a significance threshold of *p* < 0.05. Gene set enrichment analysis (GSEA) functional enrichment analysis was applied to identify significantly enriched biological processes between the different treatment groups to identify the biological functions that were significantly enriched in the different treatment groups. The composition of immune cell subpopulations in the kidney microenvironment was estimated using the CIBERSORT algorithm, and the proportions of the 22 immune cell subpopulations in each treatment group were inferred based on the standard LM22 gene signature matrix.

### Cell culture and stable infection

RAW264.7 macrophages were obtained from the American Type Culture Collection (ATCC) which provides standardized and quality-controlled cell resources. The supplier performs authentication and routine screening for Mycoplasma contamination before distribution. RAW264.7 macrophages were maintained in Dulbecco’s Modified Eagle Medium (DMEM) supplemented with 10% fetal bovine serum (Gibco). For CX3CL1 knockdown, cells were transduced with the lentiviral vector Ubi-MCS-CBh-gcGFP-IRES-Puro-CX3CL1, following the manufacturer’s protocol (Shanghai Genechem Co., Ltd.). CX3CL1/Fractalkine Recombinant protein was dissolved in water at a concentration of 46 μM, used at a concentration of 100 nM, and incubated for 24 h according to the previously established experimental protocol [17]. To inhibit TFAM and CX3CR1 expression, siRNA sequences were used. TFAM siRNA (cat. no. SC-45912; Santa Cruz Biotechnology, Inc.) and CX3CR1 siRNA (cat. no. sc-39905; Santa Cruz Biotechnology, Inc.) were used to transfect cells. To simulate the AKI inflammatory environment in vitro, RAW264.7 cells were stimulated with 1 μg/mL LPS for 12 h. Each group of cells has three replicates (*n* = 3).

### Histopathological evaluation of kidney injury

Kidney tissues were fixed in formalin, embedded in paraffin, and sectioned at a thickness of 4 μm for hematoxylin and eosin (H&E) and periodic acid-Schiff (PAS) staining. Tubular injury was assessed in a blinded manner and scored based on the percentage of damaged tubules as follows: 0, no injury; 1, <25%; 2, 25–50%; 3, 50–75%; and 4, >75%. For each mouse, at least five randomly selected fields were evaluated, and the mean score was used to quantify renal tubular injury. Histological scoring was performed in a blinded manner.

### MDA analysis

The contents of malondialdehyde (MDA) in kidney tissues were detected using MDA assay kits (thiobarbituric acid method, Jiancheng Bioengineering Institute, Nanjing, China), following the guidelines of the manufacturers of the respective kits.

### Detection of renal reactive oxygen species (ROS) by fluorescence microscopy

Intrarenal ROS levels were assessed using the 20 μM dihydroethidium (DHE-DA; Meilunbio, Dalian, China). Following the manufacturer’s protocol, stained kidney tissues were visualized under a fluorescence microscope (Olympus, Tokyo, Japan) to determine ROS production.

### Immunofluorescence staining for inflammatory and mitochondrial markers

For both cultured cells and kidney tissue sections, fixation was performed using 4% paraformaldehyde in PBS for 15 min, followed by permeabilization with 0.1% Triton X-100 for an additional 15 min. Samples were incubated overnight at 4 °C with primary antibodies using the TSA kit protocol. Antibodies were applied at the following dilutions: anti-TNF-α (1:200), anti-NGAL (1:20), anti-Kim-1 (1:200), anti-CD86 (1:200), anti-CD206 (1:200), anti-iNOS (1:200), anti-IL-10 (1:200), anti-AQP1 (1:200), anti-CX3CL1 (1:100), anti-TOM20 (1:50), anti-SOD2 (1:200), anti-Mfn2 (1:200), anti-PGC-1α (1:200), anti-DRP1 (1: 200), anti-TFAM (1:200), anti-dsDNA (1: 50), and anti-Bax (1:50). Fluorescence imaging was conducted using an FV3000 confocal laser scanning microscope (Olympus, Tokyo, Japan).

### Quantitative real-time PCR (qRT-PCR) analysis of gene expression

Total RNA was isolated from kidney tissues and RAW264.7 cells using TRIzol reagent (Invitrogen, USA). The QuantiNova SYBR Green PCR Kit (QIAGEN, Germany) was used for quantitative PCR analysis. Primer sequences are provided in Table S1. GAPDH served as the internal reference gene, and relative expression levels were calculated using the 2^−ΔΔCt^ method.

### ELISA

Collect the supernatant from all samples. Determine the levels of these cytokines using ELISA kits (including the TNF-α kit (Cat No. MTA00B), IL-1β kit (Cat No. MLB00C), IL-6 kit (Cat No. M6000B), and IL-10 kit (Cat No. M1000B)) according to the manufacturer’s (R&D Systems) protocol manual. Plot a standard curve for each cytokine and measure antigen concentration at 450 nm wavelength.

### Transmission electron microscopy for mitochondrial ultrastructure

For ultrastructural analysis, 1 mm³ kidney tissue samples were fixed in TEM fixative at 4 °C for 2 h. The samples were subsequently processed into ultrathin sections (~60 nm), stained with uranyl acetate and lead citrate, and examined using a transmission electron microscope (HT7700, HITACHI, Japan).

### Western blot analysis of protein expression in kidney and macrophages

Kidney tissues and RAW264.7 cells were lysed in radioimmunoprecipitation assay (RIPA) buffer supplemented with protease inhibitors, followed by incubation on ice for 20 min. Protein concentrations were quantified, and 50 μg of total protein per sample was separated by SDS-PAGE. Proteins were transferred to membranes and probed with the following primary antibodies: anti-CX3CL1 (1:1000), anti-NGAL (1:2000), anti-Kim-1 (1:1000), anti-TNF-α (1:500), anti-CD206 (1:1000), anti-IL-10 (1:1000), anti-CD86 (1:1000), anti-iNOS (1:1000), anti-PGC-1α (1:1000), anti-Mfn2 (1:1000), anti-DRP1 (1:1000), anti-Fis1 (1:1000), anti-TFAM (1:5000), anti-Tomm20 (1:200), anti-SOD2 (1:1000), anti-ATP5A (1:200), anti-ATPB (1:200), anti-NDUFB8 (1:5000), anti-SDHB (1:5000), anti-MTCO1 (1:1000), anti-UQCRC2 (1:2000), anti-Bax (1:200), anti-cGAS (1:5000), anti-STING (1:10000), anti-p-TBK1 (1:1000), anti-TBK1 (1:1000), anti-p-IRF3 (1:1000), and anti-IRF3 (1:1000).

### Immunohistochemical detection of protein expression in kidney tissues

Paraffin-embedded kidney sections were dewaxed and subjected to antigen retrieval. After blocking with 5% bovine serum albumin (BSA), sections were incubated with appropriate primary and secondary antibodies. Diaminobenzidine (DAB) was used for signal detection, followed by counterstaining with hematoxylin. Images were acquired using an Olympus microscope (Japan) and quantitatively analyzed.

### Mitochondrial functional assays in RAW264.7 macrophages

To assess mitochondrial function, RAW264.7 cells were stained with MitoTracker Deep Red FM, MitoSOX, mitochondrial permeability transition pore (mPTP) dye, and tetramethylrhodamine methyl ester (TMRM), following the respective manufacturer’s protocols. Mitochondrial signals were visualized and analyzed using a confocal laser scanning microscope (FV3000, Olympus, Tokyo, Japan).

### Mitochondrial respiratory enzymatic activities assay

RAW264.7 macrophages were harvested using a commercially available kit and assayed for complex I-V using commercially available kits (Cat: BC0515, BC3235, BC3245, BC0945, and BC1445, Solarbio, Beijing, China) according to the manufacturer’s protocol.

### Seahorse assay

Mitochondrial respiratory capacity was assessed in RAW264.7 macrophages using an Agilent Seahorse XF24 extracellular flux analyzer (Agilent Technologies, Inc.) RAW264.7 cells were inoculated at a density of 1 × 10^4^ per well and cultured in XF24 plates. Cells were placed in the machine according to the protocol provided by Agilent Technologies (https://agilent.com.cn) and OCR was measured by successive supplementation with oligomycin A (1 μM), carbonyl cyanide *p*-trifluoromethoxyphenylhydrazone (FCCP; 1 μM), and rotenone/antimycin A (0.5 μM). Oxygen consumption rate was measured after the addition of these drugs.

### Statistical analysis

All data are presented as mean ± standard deviation (SD) (*n* = 3–6 repeats/group). Data distribution was assessed for normality and variance homogeneity before applying parametric tests. For comparisons among three or more groups, one-way ANOVA followed by Tukey’s post hoc test was used. For transcriptomic data or datasets not meeting normality assumptions, the non-parametric Kruskal–Wallis test was applied. All tests were two-sided, and adjustments for multiple comparisons were performed where appropriate. Statistical analyses were conducted using GraphPad Prism (version 8.0, Dotmatics) and R (version 4.3.1). *P* < 0.05 was considered to indicate a statistically significant difference.

## Results

### CX3CL1 deficiency preserves renal function and attenuates pathological injury in LPS-induced AKI mice

**Fig. 1:**
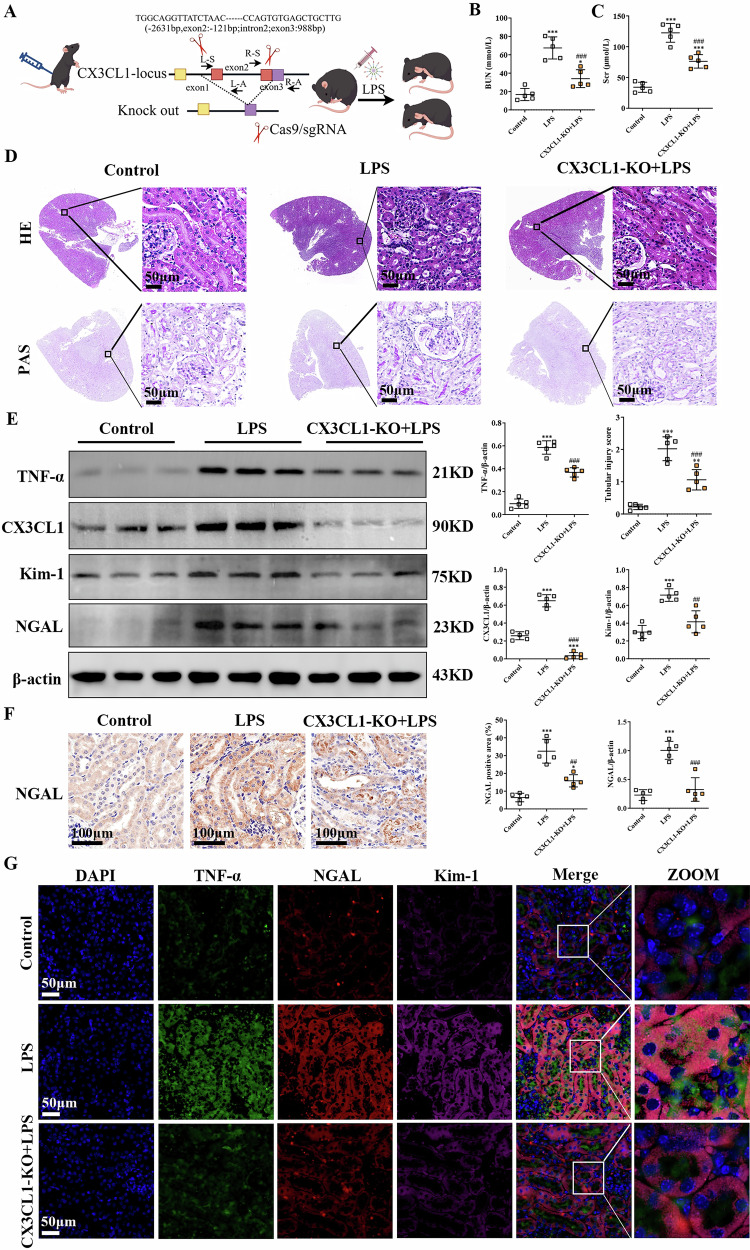
CX3CL1 knockout inhibits kidney dysfunction and inflammation in LPS-induced AKI mice. **A** Schematic of the strategy for generating CX3CL1-knockout inflammatory model mice. **B**, **C** Serum creatinine (Scr) and blood urea nitrogen (BUN) levels in kidney tissues. **D** Representative images of H&E and PAS staining (scale bar = 50 μm), and quantification of tubular injury scores in kidney tissues. **E** Western blot analysis of TNF-α, CX3CL1, NGAL, and Kim-1 protein expression in kidney tissues. **F** Immunohistochemistry for NGAL expression in kidney tissues (scale bar = 100 μm). **G** Immunofluorescence analysis showing the expression and localization of TNF-α, Kim-1, and NGAL in kidney tissues (scale bar = 50 μm). *p < 0.05, **p < 0.01, ***p < 0.001 vs control group. ^#^*p* < 0.05, ^##^*p* < 0.01, ^###^*p* < 0.001 vs LPS group.

In LPS-induced AKI mice, significant increases in Scr and BUN levels were observed, along with evident histological abnormalities in the kidney, including tubular epithelial cell necrosis and cell loss, accompanied by increased expression of kidney injury markers Kim-1 and NGAL, as well as the pro-inflammatory cytokine TNF-α, compared to control mice. However, reduced CX3CL1 expression markedly improved kidney function, as reflected by reductions in Scr and BUN levels, and also led to decreased protein expression levels of Kim-1 and NGAL, alongside attenuation of the kidney inflammatory response in CX3CL1-deficient AKI mice relative to untreated AKI mice (Fig. 1A–E). Furthermore, immunohistochemical analysis revealed a substantial increase in NGAL expression in the kidneys of LPS-induced AKI mice, whereas NGAL levels were notably decreased in the CX3CL1-deficient group (Fig. 1F). Similarly, immunofluorescence assays demonstrated that the expression of TNF-α, NGAL, and Kim-1 proteins was elevated in kidney tissues of AKI mice, while CX3CL1 deficiency leads to reduced levels of TNF-α, NGAL and Kim-1 proteins in the kidney tissue of AKI mice. (Fig. 1G). These findings suggest that CX3CL1 deficiency prevents LPS-induced kidney injury and inflammation in AKI mice.

### RNA sequencing (RNA-Seq) reveals association of kidney macrophages with CX3CL1 in AKI

**Fig. 2:**
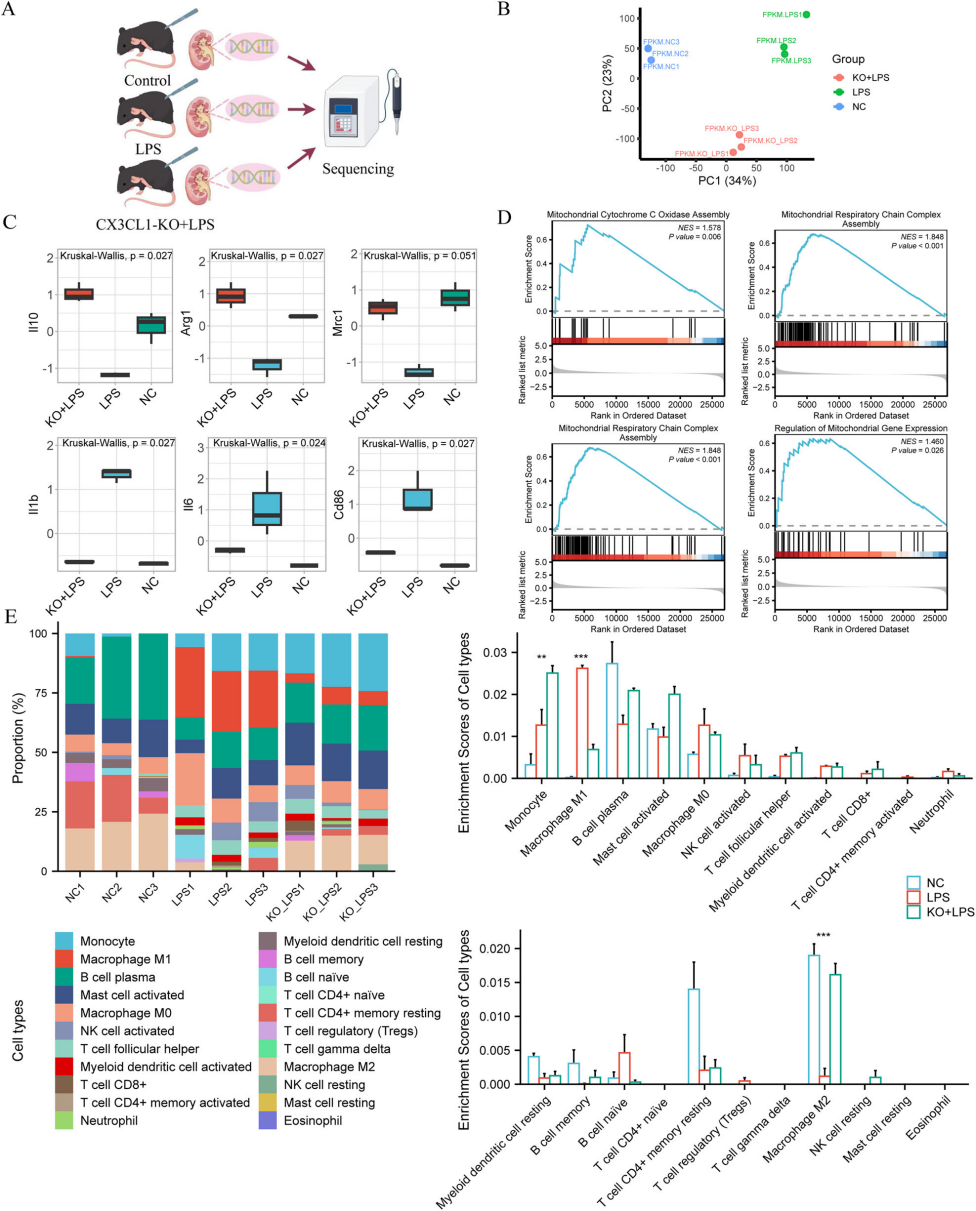
Association between kidney CX3CL1 expression and macrophage populations in LPS-induced AKI mice. **A** Workflow of transcriptome sequencing from mouse kidney tissues. **B** Principal component analysis (PCA) of RNA-Seq data from different treatment groups. **C** Gene expression profiles of il10, Arg1, Mrc1, il1b, il6, and cd86 based on transcriptome sequencing. **D** Gene set enrichment analysis (GSEA) analysis of differentially expressed genes. **E** Analysis of immune cell infiltration using the CIBERSORT algorithm. **p < 0.01, ***p < 0.001.

RNA-Seq was performed on kidney samples from the Control, LPS, and CX3CL1-knockout (KO) + LPS groups, and principal component analysis (PCA) demonstrated a clear separation among these groups at the transcriptomic level (Fig. 2A, B), indicating substantial global gene expression differences induced by LPS and modulated by CX3CL1 deficiency. Further gene expression analysis revealed that several macrophage-related genes were differentially expressed across the groups. In particular, the expression of M2-type macrophage-associated genes, including Il10, Arg1, and Mrc1, was significantly lower in both the KO + LPS and NC groups compared to the LPS group. Conversely, M1-type macrophage-associated genes such as il-1β, il-6, and cd86 exhibited significantly higher expression in the LPS group relative to the KO + LPS and NC groups (Fig. 2C). These findings suggest that CX3CL1 deletion may influence macrophage subtype composition within the kidney microenvironment during AKI. To further delineate the molecular processes affected by CX3CL1 deficiency under lipopolysaccharide (LPS)-induced inflammatory conditions, we performed differential gene expression analysis between KO-LPS and LPS groups followed by gene set enrichment analysis (GSEA). The results revealed significant enrichment of gene sets related to mitochondrial respiratory chain complex assembly, cytochrome c oxidase assembly, and regulation of mitochondrial gene expression (Fig. 2D). Moreover, immune cell infiltration analysis using the CIBERSORT algorithm revealed that CX3CL1 deficiency significantly altered the immune landscape in LPS-induced kidneys, particularly in macrophage populations. The LPS group showed increased infiltration of M1-type macrophages and decreased infiltration of M2-type macrophages compared to the NC group. In contrast, the KO + LPS group displayed a significant reduction in M1-type macrophage infiltration alongside a marked increase in M2-type macrophage infiltration (Fig. 2E). Together, these results indicate that CX3CL1 deletion suppresses LPS-induced M1-type polarization while promoting M2-type polarization of macrophages in the AKI kidney microenvironment.

### Inhibition of CX3CL1 promotes the anti-inflammatory phenotype of macrophages in LPS-induced AKI mice

**Fig. 3:**
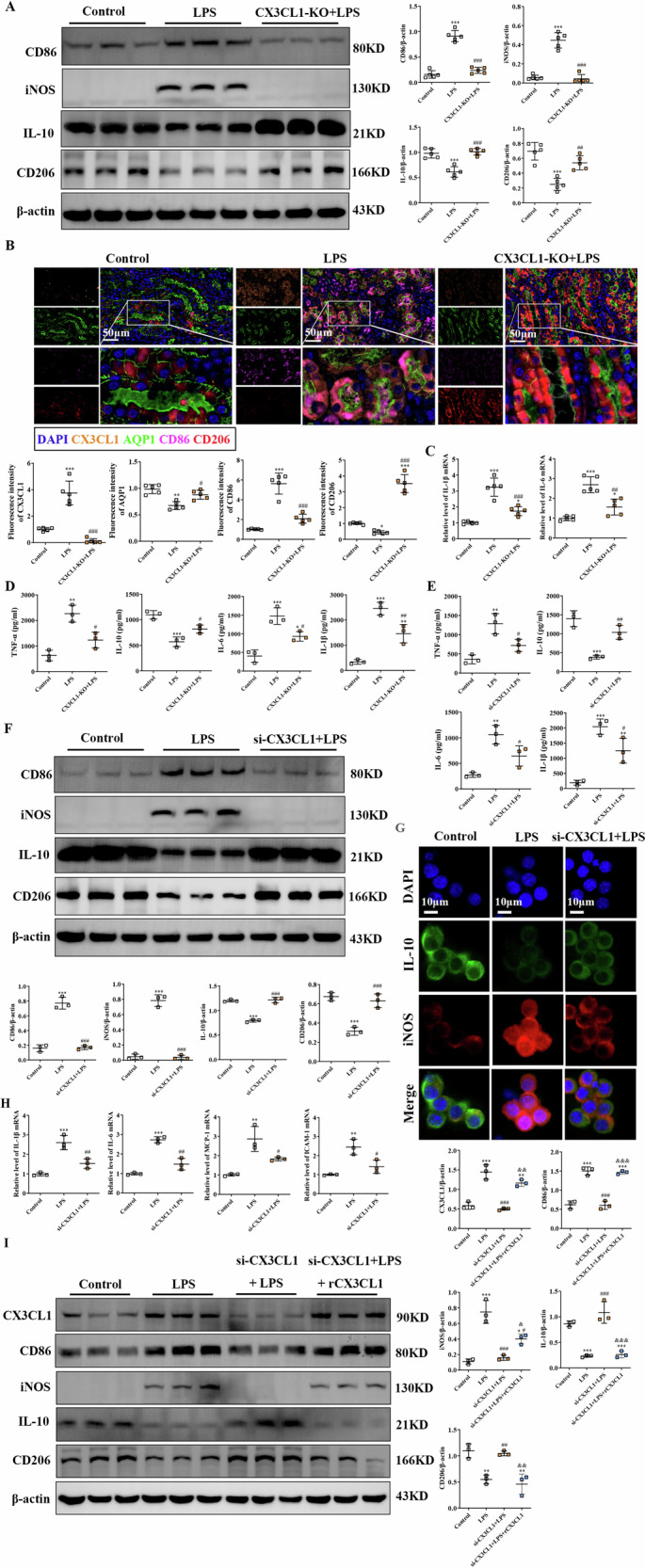
CX3CL1 deficiency converts LPS-induced M1-type macrophages to M2-type. **A** Western blot analysis of iNOS, CD86, CD206, and IL-10 protein expression in mouse kidney tissues. **B** Immunofluorescence detection of CX3CL1, AQP1, CD86, and CD206 protein localization and expression in mouse kidney tissues (scale bar = 50 μm). **C** qRT-PCR analysis of IL-1β and IL-6 mRNA levels in kidney tissues. **D**, **E** ELISA assay was used to detect the content of TNF-α, IL-6, IL-1β, and IL-10 in the mouse serum and cell supernatant. **F** Western blot analysis of iNOS, CD86, CD206, and IL-10 protein expression in RAW264.7 macrophages. **G** Immunofluorescence staining for iNOS and IL-10 in RAW264.7 macrophages (scale bar = 10 μm). **H** qRT-PCR analysis of IL-1β, IL-6, MCP-1, and ICAM-1 mRNA levels in RAW264.7 macrophages. **I** The expressions of CX3CL1, iNOS, CD86, CD206, and IL-10 protein were detected by Western blot assay. *p < 0.05, **p < 0.01, ***p < 0.001 vs control group. ^#^*p* < 0.05, ^##^*p* < 0.01, ^###^*p* < 0.001 vs LPS group. ^&^*p* < 0.05, ^& &^*p* < 0.01, ^& & &^*p* < 0.001 vs si-CX3CL1 + LPS group.

To further investigate the effect of CX3CL1 inhibition on renal macrophages, we examined the expression of characteristic marker proteins associated with M1 (pro-inflammatory) and M2 (anti-inflammatory) phenotypes. In LPS-induced AKI mice, the expression levels of M1 markers, including CD86, iNOS, and the inflammatory cytokines TNF-α, IL-1β and IL-6, were significantly increased. Additionally, CX3CL1 was highly localized in the renal tubular epithelium, confirming its involvement in inflammatory activation (Fig. 3A–D). However, inhibition of CX3CL1 shifted macrophage polarization from the M1 to the M2 phenotype, as evidenced by decreased expression of CD86, iNOS, TNF-α, IL-1β, and IL-6, accompanied by increased expression of the M2-associated markers CD206 and IL-10, with the in vitro experiments using LPS-treated RAW264.7 macrophages demonstrating similar trends. CX3CL1 knockdown promoted a shift from M1-type to M2-type polarization, as shown by western blot and immunofluorescence analyses, which revealed downregulation of CD86, iNOS, IL-1β, IL-6, MCP-1, and ICAM-1, and upregulation of CD206 and IL-10 in LPS-stimulated cells (Fig. 3E–H). Meanwhile, Western blot analysis revealed that recombinant CX3CL1 reversed the effects of CX3CL1 knockout on LPS-induced polarization in RAW264.7 macrophages. Specifically, compared to the si-CX3CL1 + LPS group, the si-CX3CL1 + LPS+rCX3CL1 group exhibited reduced CD206 and IL-10 protein levels, while CD86 and iNOS protein levels increased (Fig. 3I). These findings suggest that CX3CL1 inhibition mitigates kidney injury in AKI by promoting a phenotypic switch of macrophages from a pro-inflammatory M1 type to an anti-inflammatory M2 type, thereby reducing inflammation and pathological damage.

### Inhibition of CX3CL1 prevented LPS-induced mitochondrial injury and oxidative stress in macrophages of AKI mice

**Fig. 4:**
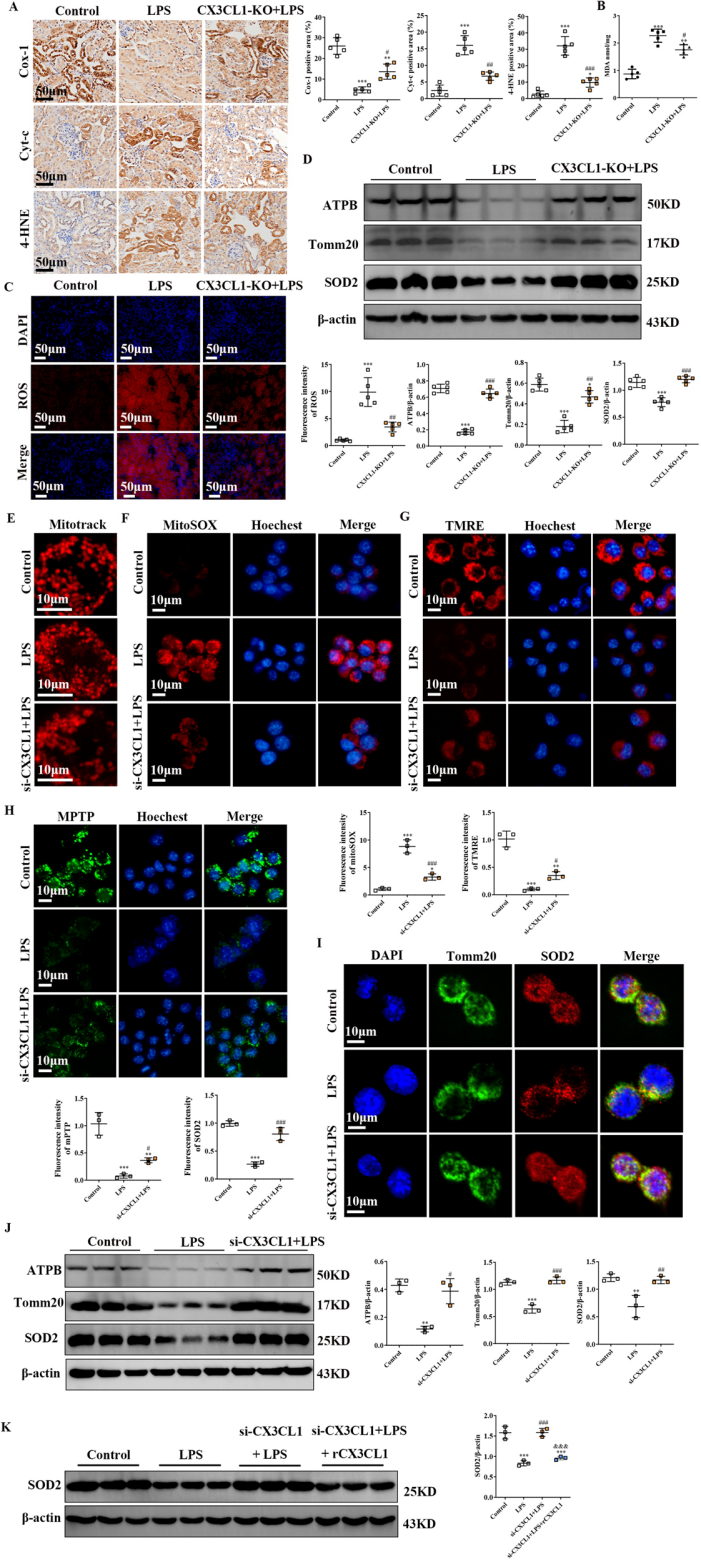
CX3CL1 deficiency alleviates LPS-induced mitochondrial injury and oxidative stress in kidney macrophages. **A** Immunohistochemical staining for Cyt-c, COX-1, and 4-HNE in mouse kidney tissues (scale bar = 50 μm). **B** Measurement of MDA levels in kidney tissues. **C** DHE staining was conducted to determine the ROS level in renal tissues. (scale bar = 50 μm). **D** Western blot analysis of Tomm20, ATPB, and SOD2 protein expression in kidney tissues. **E** Assessment of mitochondrial morphology in RAW264.7 macrophages using MitoTracker staining (scale bar = 10 μm). **F** MitoSOX staining to detect mitochondrial ROS in RAW264.7 macrophages (scale bar = 10 μm). **G**, **H** Evaluation of mitochondrial membrane potential using TMRE and mPTP staining in RAW264.7 macrophages (scale bar = 10 μm). **I** Immunofluorescence analysis of SOD2 and Tomm20 expression in RAW264.7 macrophages (scale bar = 10 μm). **J** Western blot analysis of ATPB, SOD2, and Tomm20 protein expression in RAW264.7 macrophages. **K** The expression of SOD2 protein was detected by Western blot in RAW264. 7 macrophages. *p < 0.05, **p < 0.01, ***p < 0.001 vs control group. ^#^*p* < 0.05, ^##^*p* < 0.01, ^###^*p* < 0.001 vs LPS group. ^&^*p* < 0.05, ^& &^*p* < 0.01, ^& & &^*p* < 0.001 vs si-CX3CL1 + LPS group.

Mitochondria, as the primary site of intracellular energy production, are also particularly susceptible to damage induced by oxidative stress, which in turn exacerbates mitochondrial dysfunction [18]. To determine whether CX3CL1 plays a regulatory role in mitochondrial injury within macrophages during AKI, we evaluated key oxidative stress-related mitochondrial proteins in vivo. Immunohistochemical analysis revealed that the expression levels of cytochrome c (Cyt-c) and cytochrome c oxidase subunit 1 (COX-1), two key mitochondrial proteins involved in electron transport and ROS generation, as well as 4-hydroxynonenal (4-HNE), a lipid peroxidation product that reflects oxidative damage, were markedly increased in the kidneys of LPS-treated mice. These elevations were significantly reduced following CX3CL1 deficiency (Fig. 4A). Moreover, CX3CL1 deficiency mitigated the LPS-induced upregulation of MDA levels (Fig. 4B). To further evaluate oxidative stress, ROS levels in kidney tissues were measured using a DHE assay. LPS stimulation led to a robust increase in ROS production, whereas CX3CL1 deficiency effectively reversed this response (Fig. 4C). The above results confirm that CX3CL1 deficiency ameliorates LPS-induced oxidative stress in mouse kidneys. Moreover, Western blot analyses demonstrated that, in CX3CL1-deficient mice, the expression of ATPB, superoxide dismutase 2 (SOD2), and the mitochondrial structural marker Tomm20 was markedly enhanced under LPS challenge, indicating improved mitochondrial preservation (Fig. 4D). Next, we examined whether the protective effect of CX3CL1 inhibition against mitochondrial injury and oxidative stress could be recapitulated in vitro. MitoTracker staining in LPS-treated RAW264.7 macrophages revealed disrupted mitochondrial networks and increased fragmentation, which were notably alleviated by siRNA-mediated knockdown of CX3CL1 (Fig. 4D). Additionally, mitochondrial ROS production, assessed using the MitoSOX probe, was significantly elevated in the LPS group but was suppressed following si-CX3CL1 treatment (Fig. 4E). Consistent with these findings, TMRE staining demonstrated that si-CX3CL1 treatment attenuated LPS-induced mitochondrial membrane potential depolarization, while mPTP probe staining showed that abnormal mitochondrial permeability transition pore opening induced by LPS was also significantly inhibited by CX3CL1 silencing (Fig. 4F, G). Immunofluorescence staining further confirmed that mitochondrial structure in LPS-treated RAW264.7 cells was disorganized, with reduced SOD2 localization, whereas si-CX3CL1 treatment restored normal mitochondrial morphology and SOD2 distribution (Fig. 4H). Western blot analysis supported these observations, showing that si-CX3CL1 reversed the LPS-induced downregulation of ATPB, SOD2, and Tomm20 protein expression in RAW264.7 cells (Fig. 4I). Furthermore, in LPS-induced RAW264.7 macrophages, recombinant CX3CL1 reversed the elevated SOD2 protein levels caused by CX3CL1 silencing, as demonstrated by Western blot analysis (Fig. 4J). These results suggest that inhibition of CX3CL1 could attenuate mitochondrial damage and oxidative stress in macrophages of AKI mice.

### Inhibition of CX3CL1 rescues reduced mitochondrial electron transport chain complex expression and cellular oxygen consumption in AKI kidney macrophages

**Fig. 5:**
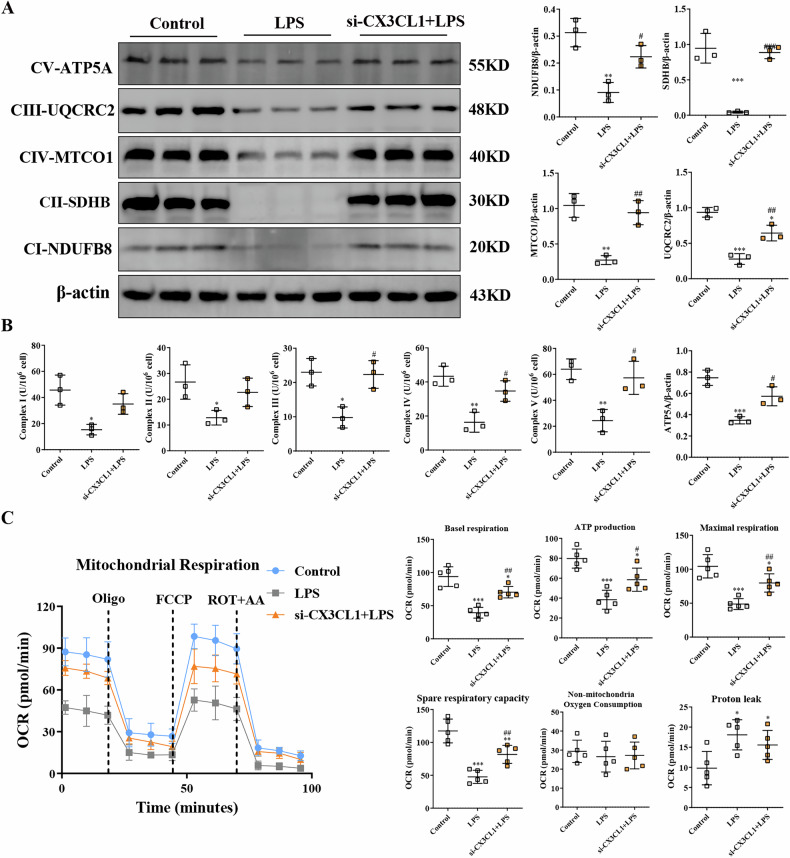
CX3CL1 regulates mitochondrial electron transport chain complex expression and cellular oxygen consumption in renal macrophages. **A** Western blot analysis of NDUFB8, SDHB, MTCO1, UQCRC2, and ATP5A protein expression in RAW264.7 macrophages. **B** Mitochondrial complexes I-V were detected in raw264.7 macrophage lysate. **C** Effect of CX3CL1 inhibition on mitochondrial respiratory capacity of LPS-induced RAW264.7 macrophages. *p < 0.05, **p < 0.01, ***p < 0.001 vs control group. ^#^*p* < 0.05, ^##^*p* < 0.01, ^###^*p* < 0.001 vs LPS group.

The electron transport chain (ETC) is a cascade of electron transport proteins that play a key role in the production of ATP by mitochondria, the main source of mitochondrial ROS. Western blot analysis showed that LPS induced a significant decrease in the expression of key subunits NDUFB8, SDHB, MTCO1, UQCRC2, and ATP5A of the mitochondrial electron transport chain complexes I-V in RAW26.4 macrophages. Inhibition of CX3CL1 elevated the expression of the ETC complex (Fig. 5A). In addition, the activity of mitochondrial complexes I-V were examined in macrophage lysates from raw264.7. LPS treatment resulted in a significant reduction in the expression levels of these complex proteins. In contrast, the inhibition of CX3CL1 attenuated this reduction (Fig. 5B). Seahorse assay showed that CX3CL1 knockdown rescued LPS-induced reductions in basal respiratory capacity, maximal respiratory capacity, ATP-producing capacity, and reserve respiratory capacity (Fig. 5C), which are indicators of mitochondrial dysfunction.

### Inhibition of CX3CL1 maintains mitochondrial fusion-fission balance and prevents mitochondrial dysfunction in AKI kidney macrophages

**Fig. 6:**
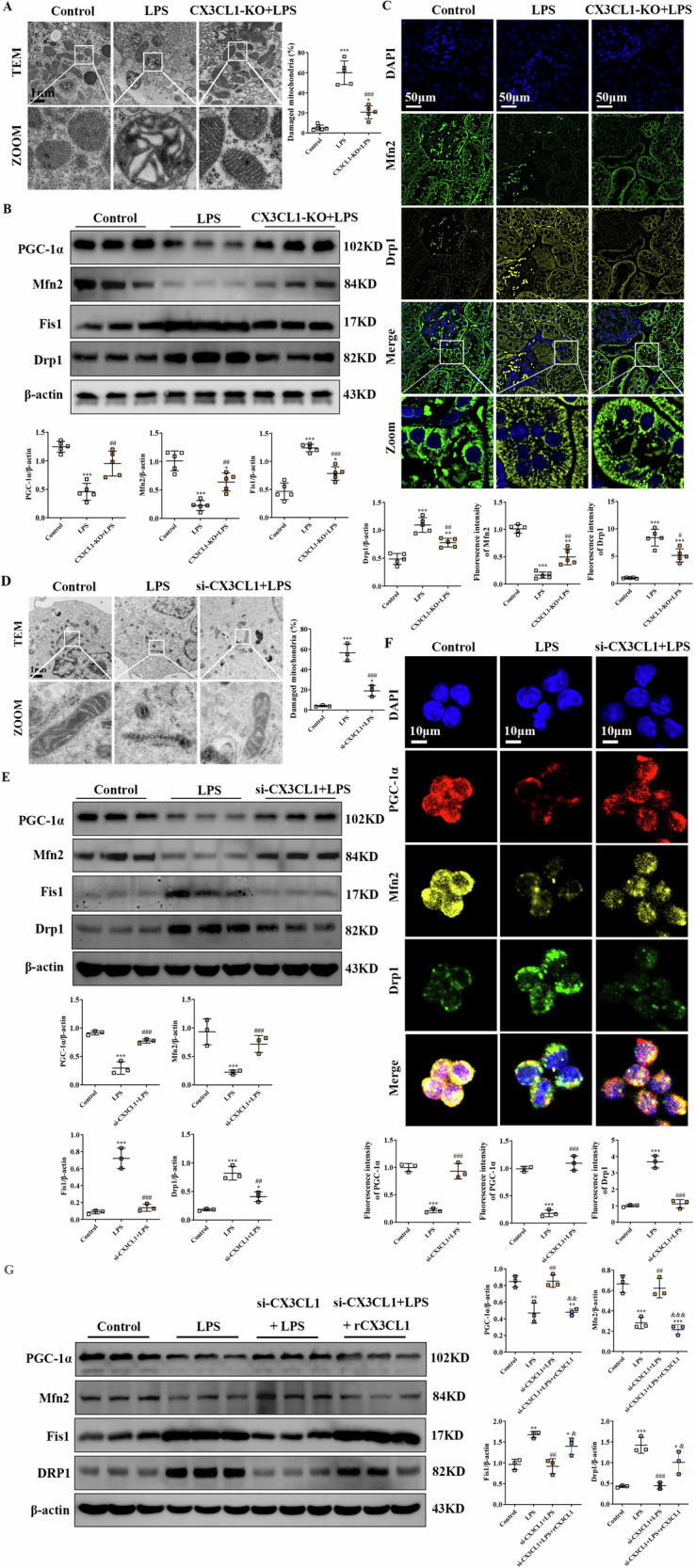
CX3CL1 deficiency maintains mitochondrial homeostasis in LPS-stimulated kidney macrophages. **A** Transmission electron microscopy reveals LPS-induced mitochondrial morphological changes in mouse kidney tissues. **B** Western blot analysis of PGC-1α, Mfn2, DRP1, and Fis1 protein expression in mouse kidney tissues. **C** Immunofluorescence analysis of Mfn2 and DRP1 expression and localization. **D** Electron microscopy showing mitochondrial ultrastructure in RAW264.7 macrophages following LPS stimulation. **E** Western blot analysis of PGC-1α, Mfn2, DRP1, and Fis1 in RAW264.7 macrophages. **F** Immunofluorescence detection of PGC-1α, Mfn2, and DRP1 expression and localization in RAW264.7 macrophages. **G** The expressions of PGC-1α, Mfn2, DRP1, and Fis1 protein were detected by Western blot in RAW264. 7 macrophages. *p < 0.05, **p < 0.01, ***p < 0.001 vs control group. ^#^*p* < 0.05, ^##^*p* < 0.01, ^###^*p* < 0.001 vs LPS group. ^&^*p* < 0.05, ^& &^*p* < 0.01, ^& & &^*p* < 0.001 vs si-CX3CL1 + LPS group.

Mitochondrial biogenesis and dynamic balance between fusion and fission play essential roles in maintaining mitochondrial populations, regulating cellular energy metabolism, and contributing to the pathogenesis of AKI [19]. To assess whether CX3CL1 inhibition prevents mitochondrial dysfunction in AKI, we examined key mitochondrial indicators both in vivo and in vitro. Electron microscopy of kidney tissues revealed that LPS-induced AKI resulted in mitochondrial structural disruption, including cristae disorganization and enlargement, whereas these ultrastructural abnormalities were markedly alleviated in CX3CL1-deficient mice (Fig. 6A). In parallel, western blot analyses demonstrated that CX3CL1 deficiency effectively restored the expression of the mitochondrial biogenesis regulator PGC-1α and the mitochondrial fusion protein Mfn2, both of which were suppressed in LPS-treated kidneys, while simultaneously reducing the elevated expression of mitochondrial fission proteins Fis1 and DRP1 (Fig. 6B). Immunofluorescence staining further supported these findings by showing that LPS diminished Mfn2 localization and increased Fis1 localization in kidney tissues, whereas these alterations were significantly reversed in CX3CL1-deficient mice (Fig. 6C). Similar protective effects of CX3CL1 inhibition were observed in LPS-treated RAW264.7 cells. Transmission electron microscopy revealed that siRNA-mediated knockdown of CX3CL1 substantially mitigated mitochondrial fragmentation induced by LPS exposure (Fig. 6D). Western blot results showed that si-CX3CL1 treatment significantly reversed the LPS-induced reduction of PGC-1α and Mfn2 protein levels and simultaneously suppressed the elevation of Fis1 and DRP1 levels (Fig. 6E). Immunofluorescence staining demonstrated enhanced localization of PGC-1α and Mfn2, along with decreased DRP1 localization in LPS-treated RAW264.7 cells following CX3CL1 silencing (Fig. 6F). Additionally, Western blot analysis revealed that recombinant CX3CL1 reversed the reduction in Fis1 and Drp1 proteins and the increase in PGC-1α and Mfn2 proteins caused by CX3CL1 silencing in LPS-induced RAW264.7 macrophages (Fig. 6G). These results demonstrate that CX3CL1 inhibition could preserve mitochondrial homeostasis by restoring the fusion-fission balance and promoting mitochondrial integrity in macrophages during AKI.

### Inhibition of CX3CL1 attenuates TFAM-related mtDNA instability in AKI kidney macrophages

**Fig. 7:**
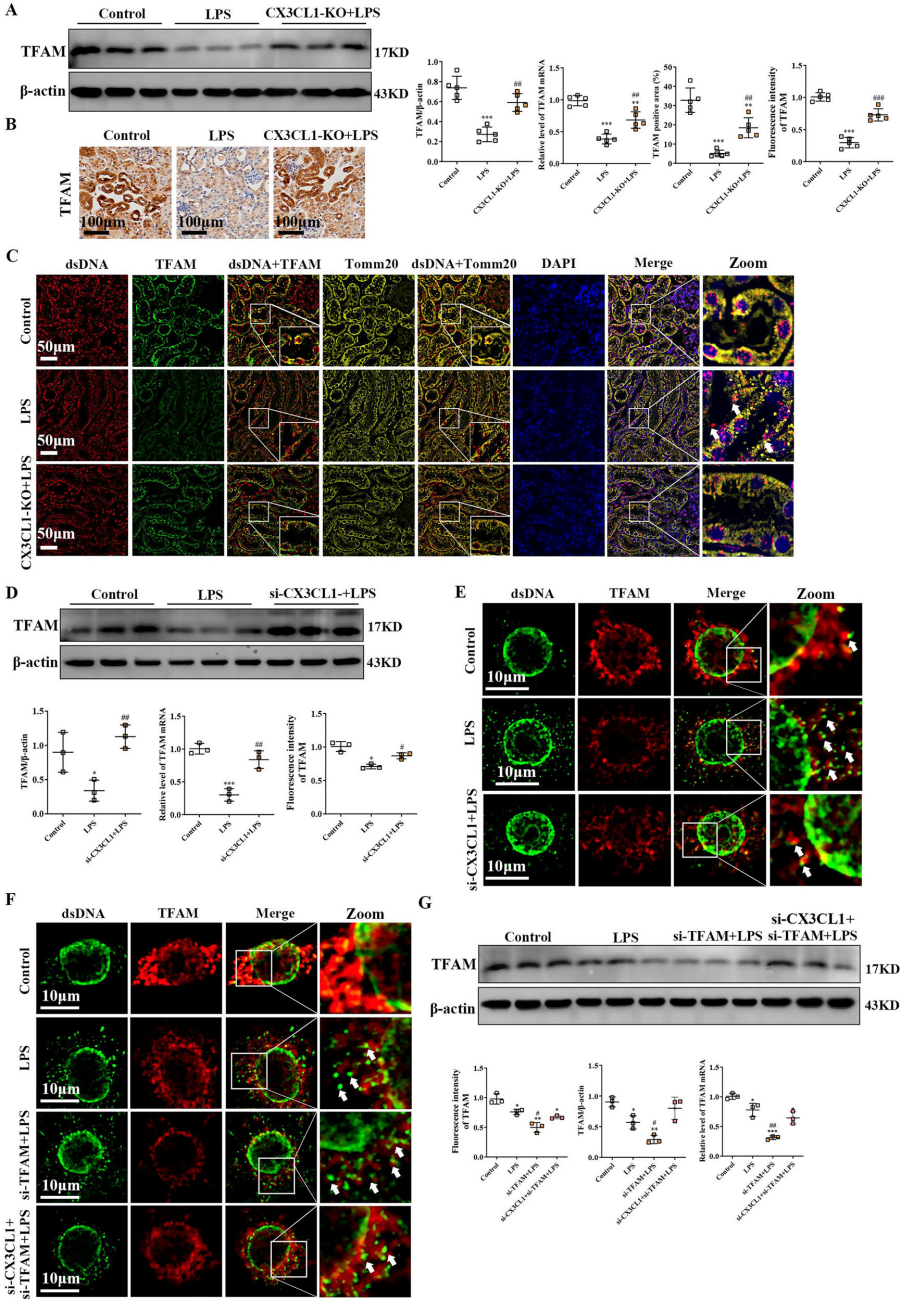
CX3CL1 deficiency attenuates LPS-induced mtDNA instability in kidney macrophages. **A** Western blot analysis of TFAM protein expression in mouse kidney tissues. **B** Immunohistochemistry to assess TFAM expression in kidney tissues (scale bar = 100 μm). **C** Immunofluorescence analysis of TFAM, dsDNA, and Tomm20 localization in mouse kidney tissues. **D** Western blot analysis of TFAM protein expression in RAW264.7 macrophages. **E** Immunofluorescence detection of TFAM and dsDNA localization in RAW264.7 macrophages. **F** Immunofluorescence analysis of TFAM and dsDNA in RAW264. 7 macrophages following si-TFAM treatment. **G** Western blot analysis of TFAM and CX3CL1 protein expression in RAW264. 7 macrophages treated with si-TFAM. *p < 0.05, **p < 0.01, ***p < 0.001 vs control group. ^#^*p* < 0.05, ^##^*p* < 0.01, ^###^*p* < 0.001 vs LPS group.

mtDNA damage is increasingly recognized as a hallmark of AKI, with mitochondrial transcription factor A (TFAM) serving as a central regulator of mtDNA maintenance and stability [20]. To investigate whether CX3CL1 modulates mtDNA stability through TFAM regulation, we examined TFAM expression and mtDNA integrity both in vivo and in vitro. Western blot and qPCR analyses revealed that CX3CL1 deficiency effectively reversed the LPS-induced suppression of TFAM protein and mRNA levels in mouse kidneys (Fig. 7A). Immunohistochemistry further confirmed that CX3CL1 knockout significantly elevated TFAM expression in kidney tissues from AKI mice (Fig. 7B). In addition, immunofluorescence analysis revealed reduced co-localization of Tomm20, TFAM, and double-stranded DNA (dsDNA) in LPS-treated kidneys, indicating impaired mtDNA stability; however, this effect was partially rescued by CX3CL1 deficiency, suggesting restored mitochondrial nucleoid integrity (Fig. 7C). Consistent findings were observed in vitro, as si-CX3CL1 treatment in LPS-stimulated RAW264.7 cells resulted in increased TFAM expression at both the protein and mRNA levels (Fig. 7D). Immunofluorescence staining in RAW264.7 cells demonstrated that LPS reduced TFAM and dsDNA co-localization, while si-CX3CL1 treatment effectively attenuated this loss (Fig. 7E). To further validate the regulatory relationship between CX3CL1 and TFAM in mediating mtDNA integrity, mtDNA leakage was examined in RAW264.7 cells using siRNA against TFAM. TFAM knockdown further enhanced LPS-induced mtDNA leakage, whereas co-treatment with si-CX3CL1 significantly reduced both LPS- and si-TFAM-induced mtDNA leakage (Fig. 7F, G). Collectively, these findings suggest that CX3CL1 inhibition enhances TFAM expression and function, thereby preserving mtDNA stability and limiting mitochondrial injury in AKI macrophages.

### Inhibition of CX3CL1 upregulated BAX and suppressed cGAS-STING-mediated inflammatory signaling in AKI kidney macrophages

**Fig. 8:**
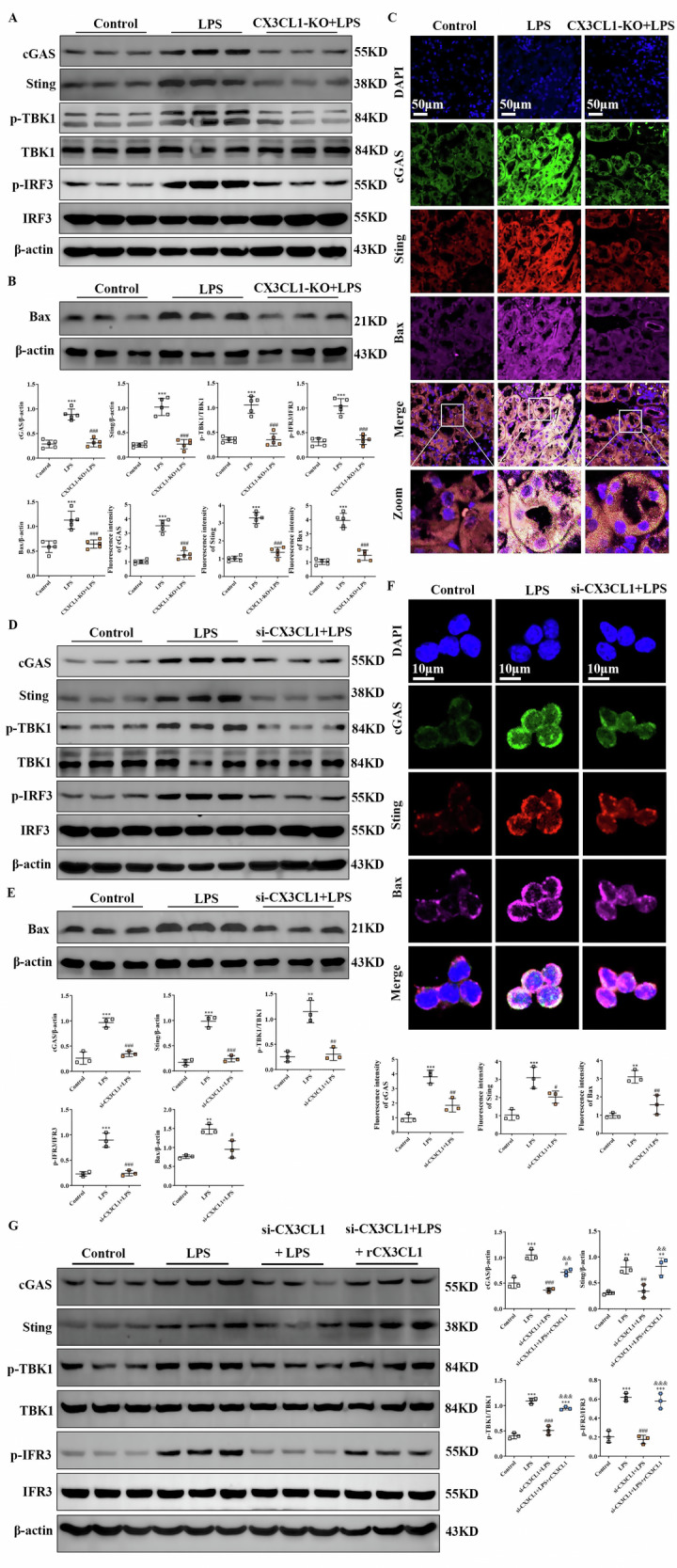
CX3CL1 deficiency inhibits activation of Bax and the cGAS-STING signaling axis in LPS-induced macrophages. **A**, **B** Western blot analysis of cGAS, STING, p-TBK1, TBK1, p-IRF3, IRF3, and Bax protein expression in mouse kidney tissues. **C** Immunofluorescence detection of cGAS, STING, and Bax expression and localization in kidney tissues (scale bar = 50 μm). **D**, **E** Western blot analysis of cGAS, STING, p-TBK1, TBK1, p-IRF3, IRF3, and Bax in RAW264. 7 macrophages. **F** Immunofluorescence staining of cGAS, STING, and Bax in RAW264. 7 macrophages (scale bar = 10 μm). **G** The expressions of cGAS, STING, p-TBK1, TBK1, p-IRF3, IRF3 protein were detected by Western blot in RAW264. 7 macrophages. *p < 0.05, **p < 0.01, ***p < 0.001 vs control group. ^#^*p* < 0.05, ^##^*p* < 0.01, ^###^*p* < 0.001 vs LPS group. ^&^*p* < 0.05, ^& &^*p* < 0.01, ^& & &^*p* < 0.001 vs si-CX3CL1 + LPS group.

Mitochondrial dysfunction increases the permeability of the outer mitochondrial membrane, facilitating mtDNA release into the cytoplasm via BAX, a process that activates the cGAS-STING pathway and contributes to the progression of AKI [21]. To explore whether CX3CL1 modulates this signaling axis, we compared control and CX3CL1-knockout (CX3CL1-KO) mice. In AKI mice, both BAX expression and the activation of the cGAS-STING pathway were significantly increased, as evidenced by the upregulation of cGAS, STING, phosphorylated TBK1 (p-TBK1), and phosphorylated IRF3 (p-IRF3) proteins. However, knockdown of CX3CL1 markedly suppressed the activation of BAX and the cGAS-STING pathway in these mice (Fig. 8A, B). Similar findings were observed in LPS-treated RAW264.7 macrophages, where siRNA-mediated silencing of CX3CL1 significantly reduced the expression of BAX and key cGAS-STING signaling components, including cGAS, STING, p-TBK1 and p-IRF3 (Fig. 8D, E). Furthermore, Immunofluorescence staining revealed enhanced co-localization of BAX, cGAS, and STING proteins in kidney tissues of AKI mice and in LPS-induced RAW264.7 cells, whereas CX3CL1 inhibition effectively attenuated this co-localization (Fig. 8C, F). Western blot analysis demonstrated that recombinant CX3CL1 reversed the reduction in cGAS, Sting, p-TBK1 and p-IRF3 protein levels induced by CX3CL1 silencing in LPS-treated RAW264.7 macrophages (Fig. 8G).

### Inhibition of CX3CR1 promotes the anti-inflammatory phenotype and alleviates mitochondrial damage in LPS-induced RAW264.7

**Fig. 9:**
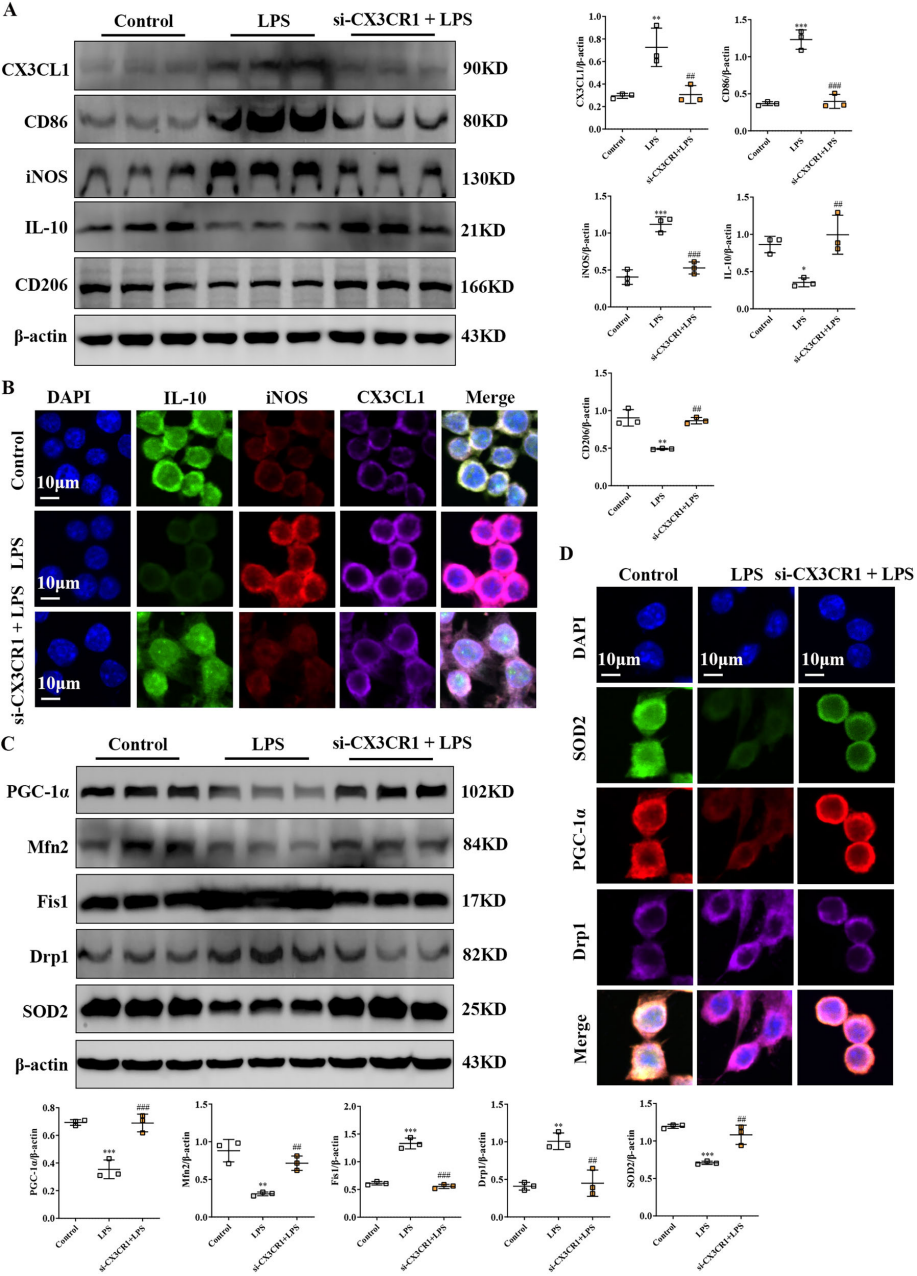
CX3CR1 silencing promotes M2 polarization and preserves mitochondrial homeostasis in LPS-stimulated macrophages. **A** Western blot analyses of CX3CL1, CD86, iNOS, IL-10, CD206 protein expression in RAW264.7 macrophage. **B** Immunofluorescence detection of CX3CL1, iNOS, and IL-10 localization in RAW264.7 macrophage (scale bar = 10 μm). **C** Western blot analyses of PGC-1α, Mfn2, Drp1, Fis1, and SOD2 protein expression in RAW264.7 macrophage. **D** Immunofluorescence staining of PGC-1α, Drp1, and SOD2 in RAW264. 7 macrophages (scale bar = 10 μm). *p < 0.05, **p < 0.01, ***p < 0.001 vs control group. ^#^*p* < 0.05, ^##^*p* < 0.01, ^###^*p* < 0.001 vs LPS group.

As a classic chemokine, the biological effects of CX3CL1 are primarily mediated through its receptor CX3CR1 [22]. To further elucidate the role of CX3CR1 in CX3CL1-mediated macrophage polarization and mitochondrial homeostasis regulation, we performed a functional knockout experiment using CX3CR1-specific siRNA in RAW264.7 macrophages. Western blot and immunofluorescence analyses revealed that CX3CR1 silencing in LPS-induced RAW264.7 macrophages reduced CD86 and iNOS expression while upregulating CD206 and IL-10, indicating a shift toward the anti-inflammatory M2 phenotype (Fig. 9A, B). Concurrently, we examined the effects of CX3CR1 knockdown on mitochondrial regulatory and oxidative stress proteins. Western blot and immunofluorescence analyses revealed that si-CX3CR1 intervention effectively reversed the significant downregulation of PGC-1α, Mfn2, and SOD2 induced by LPS exposure, while simultaneously upregulating the fission-related proteins Drp1 and Fis1 (Fig. 9C, D). Collectively, CX3CR1 inhibition not only promotes macrophage polarization toward the M2 phenotype but also mitigates LPS-induced mitochondrial damage, supporting a critical role for the CX3CL1/CX3CR1 axis in macrophage dysfunction during acute kidney injury.

### Essential role of TFAM in CX3CL1-deficiency-mediated macrophage polarization and mitochondrial function regulation

**Fig. 10:**
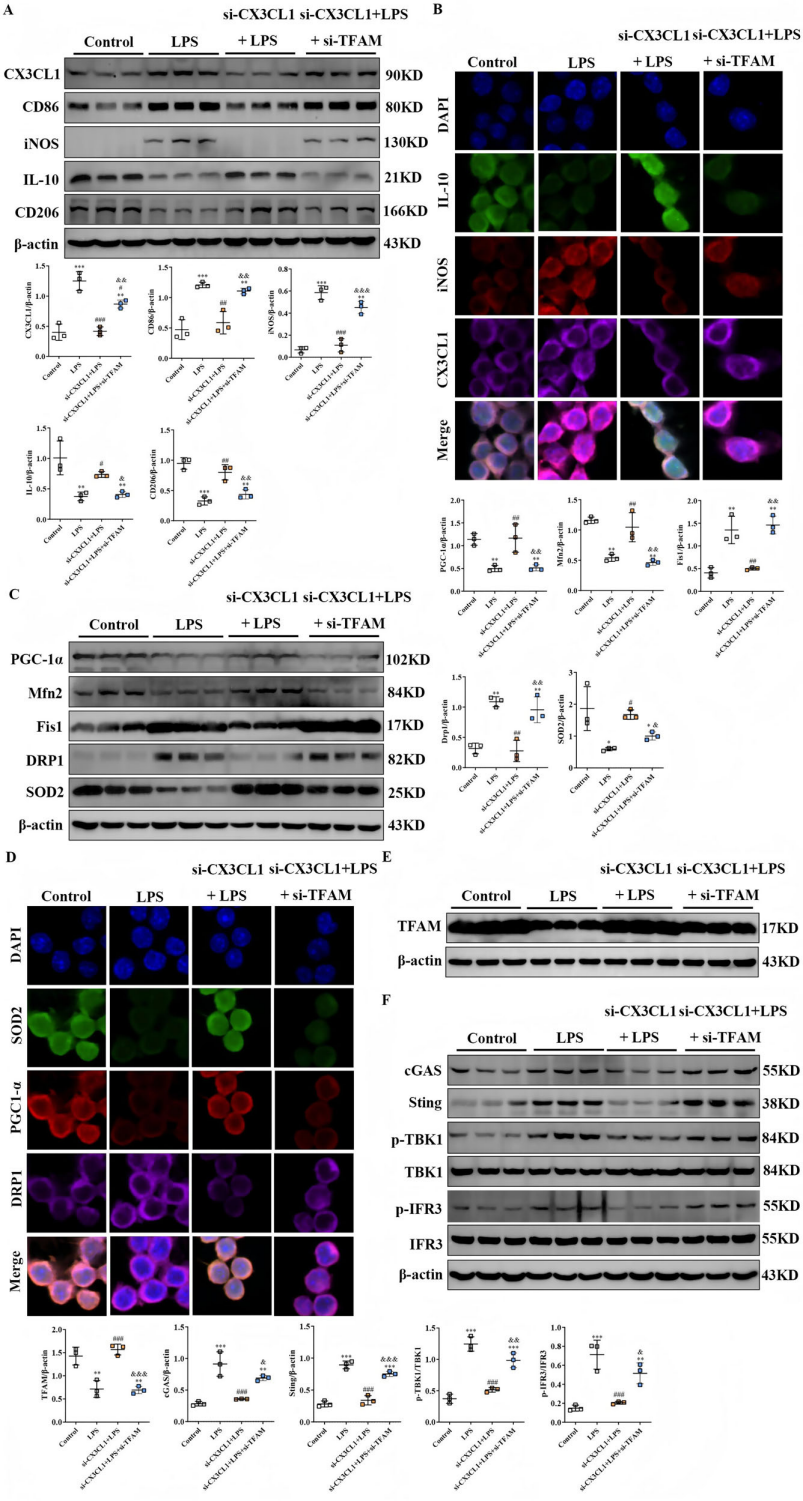
TFAM is required for CX3CL1 silencing–mediated anti-inflammatory, mitochondrial, and cGAS-STING regulatory effects in LPS-stimulated macrophages. **A** Western blot analyses of CX3CL1, CD86, iNOS, IL-10, CD206 protein expression in RAW264.7 macrophage. **B** Immunofluorescence detection of CX3CL1, iNOS, and IL-10 localization in RAW264.7 macrophage (scale bar = 10 μm). **C** Western blot analyses of PGC-1α, Mfn2, Drp1, Fis1, and SOD2 protein expression in RAW264.7 macrophage. **D** Immunofluorescence staining of PGC-1α, Drp1, and SOD2 in RAW264. 7 macrophages (scale bar = 10 μm). **E** Western blot analyses of TFAM protein expression in RAW264.7 macrophage. **F** Western blot analyses of cGAS, STING, p-TBK1, TBK1, p-IRF3, IRF3 protein expression in RAW264.7 macrophage. *p < 0.05, **p < 0.01, ***p < 0.001 vs control group. ^#^*p* < 0.05, ^##^*p* < 0.01, ^###^*p* < 0.001 vs LPS group. ^&^*p* < 0.05, ^& &^*p* < 0.01, ^& & &^*p* < 0.001 vs si-CX3CL1 + LPS group.

To determine whether TFAM is indispensable for the protective effects conferred by CX3CL1 inhibition, we silenced TFAM expression in RAW264.7 macrophages under LPS stimulation. Western blot and immunofluorescence analyses revealed that LPS significantly upregulated CD86 and iNOS while downregulating CD206 and IL-10. CX3CL1 knockdown significantly reversed LPS effects, manifesting as decreased CD86 and iNOS levels and increased CD206 and IL-10 levels. However, simultaneous TFAM knockdown largely eliminated these effects, restoring the LPS-induced M1 phenotype (Fig. 10A, B). We concurrently examined mitochondrial regulatory proteins. LPS stimulation suppressed PGC-1α and Mfn2 expressions while upregulating Drp1 and Fis1. CX3CL1 silencing reversed these abnormalities, but additional TFAM knockdown abrogated the improvement, reinstating an LPS-like mitochondrial dysfunction pattern. Furthermore, the oxidative stress marker SOD2 was maintained under CX3CL1 inhibition but significantly decreased in the si-CX3CL1+si-TFAM group (Fig. 10C, D). We further assessed cGAS-STING pathway activation by Western blot assay. LPS strongly induced upregulation of cGAS, STING, p-TBK1, and p-IRF3, an effect significantly suppressed in CX3CL1-silenced macrophages. Crucially, TFAM knockdown restored cGAS-STING pathway activation in the CX3CL1-deficient context, indicating that TFAM-mediated mitochondrial stabilization is essential for suppressing this pathway (Fig. 10E). Collectively, these findings reveal TFAM as a key mediator of anti-inflammatory, mitochondrial protective, and cGAS-STING inhibitory effects in CX3CL1-deficient macrophages, highlighting the central role of the CX3CL1/TFAM axis in regulating macrophage function during inflammatory stress.

## Discussion

Research on the role of CX3CL1 in AKI is motivated by the high incidence and mortality associated with this condition, along with the lack of effective therapeutic strategies. Although existing literature has implicated CX3CL1 in inflammation and apoptosis [23], its precise role in AKI remains insufficiently characterized. The present study addresses this gap by elucidating the function of CX3CL1 in modulating macrophage behavior within the kidney microenvironment, offering insights into its therapeutic potential. Our results demonstrate that CX3CL1 deficiency significantly improves kidney function in AKI mouse models, attenuates inflammatory responses, and promotes a phenotypic shift in macrophages from a pro-inflammatory M1 type to an anti-inflammatory M2 type. In addition, CX3CL1 inhibition was shown to alleviate mitochondrial dysfunction and reduce mtDNA leakage, thereby contributing to the overall mitigation of kidney injury. These findings collectively support the value of CX3CL1 both as a potential biomarker and a therapeutic target in AKI and lay the foundation for future research into targeted interventions for this serious clinical condition.

Leukocytes play a key role in AKI pathogenesis by migrating and differentiating within injured renal tissues under the regulation of chemokines [24]. Among these, CX3CL1 has attracted attention due to its high expression in kidney disease. Unlike other chemokines, CX3CL1 functions dually as a chemokine and an adhesion molecule, enabling it not only to recruit leukocytes, particularly monocytes and macrophages, but also to mediate direct interactions between damaged renal parenchymal cells and infiltrating immune cells [25]. Previous studies have supported its pathogenic role: for instance, Oh et al. reported that CX3CL1 inhibition conferred protection against ischemia-induced acute renal failure [26], while Song et al. showed that CX3CL1 inhibition attenuated extracellular matrix accumulation in a mouse model of diabetic nephropathy [27]. These findings suggest the potential of inhibiting CX3CL1 for the treatment of kidney disease. In our study, we confirmed that CX3CL1 expression was elevated in the kidneys of AKI mice. CX3CL1 gene deletion significantly reduced the deterioration of kidney function, tubular injury, and elevated inflammatory factors in AKI mice. These findings suggest that inhibition of CX3CL1 in kidney tissues may be a potential target for reducing inflammation and kidney injury.

The role of macrophages in AKI has garnered increasing attention due to their dynamic and multifaceted functions in injury, inflammation, and repair [28]. Unlike resident kidney cells, such as podocytes, tubular epithelial cells, and mesangial cells, which contribute to structural and functional integrity, macrophages exhibit remarkable plasticity and play a central role in orchestrating immune responses, tissue repair, and fibrosis [29]. This distinct characteristic makes them a compelling focus in AKI research. One key advantage of studying macrophages over intrinsic kidney cells lies in their dual role in both injury progression and resolution. While resident cells such as tubular epithelial cells are primarily passive responders to ischemic or toxic insults, macrophages actively modulate the injury microenvironment through phenotypic polarization into M1 (pro-inflammatory) and M2 (anti-inflammatory) subsets [30]. This polarization enables macrophages to either exacerbate tissue injury or promote tissue repair, making them an attractive target for therapeutic intervention. In line with this, our findings demonstrate that CX3CL1 deficiency plays a key role in modulating macrophage polarization, significantly shifting the balance from the pro-inflammatory M1 phenotype to the anti-inflammatory M2 phenotype, which is essential for mitigating AKI, as evidenced by the reduced expression of M1 markers such as CD86 and iNOS, along with elevated expression of M2 markers including CD206 and IL-10. The observed modulation of macrophage phenotype highlights the role of CX3CL1 in regulating inflammatory responses and underscores its contribution to the enhancement of renal recovery. Given that the transition from M1 to M2 macrophages is central to kidney repair processes, and that M2 macrophages are known to secrete anti-inflammatory cytokines and support tissue regeneration, the ability of CX3CL1 inhibition to promote this shift provides a promising therapeutic strategy. Therefore, facilitating an anti-inflammatory environment through CX3CL1 suppression may mitigate tissue damage and enhance functional recovery in AKI.

Mitochondrial integrity is crucial for maintaining kidney cellular energetics, redox balance, and apoptosis regulation [31]. In AKI, mitochondrial damage results in ATP depletion, excessive production of ROS, and the release of pro-apoptotic factors, which contribute to the exacerbation of kidney injury [32]. This study showed that LPS-induced AKI was associated with impaired mitochondrial biogenesis, disrupted mitochondrial dynamics, and increased oxidative stress. Specifically, the expression of PGC-1α, a key regulator of mitochondrial biogenesis, and Mfn2, a major mitochondrial fusion protein, was reduced, whereas levels of DRP1, a mitochondrial fission protein, along with oxidative stress markers such as Cyt-c, COX-1, and 4-HNE, were elevated. Additionally, mitochondrial ultrastructure appeared disorganized, and membrane potential was significantly reduced. Importantly, genetic deletion of CX3CL1 effectively reversed these mitochondrial abnormalities, indicating that CX3CL1 plays a pivotal role in mediating mitochondrial dysfunction and oxidative damage during AKI. Given the important contribution of mitochondrial impairment to AKI pathogenesis, therapeutic strategies aimed at enhancing mitochondrial biogenesis or restoring the balance of mitochondrial fusion and fission represent promising approaches. Pharmacological agents such as SS-31, a mitochondria-targeted peptide, and MitoQ, a mitochondrial antioxidant, have shown protective effects in preclinical AKI models by preserving mitochondrial function and attenuating oxidative stress [33, 34]. Our findings suggest that inhibition of CX3CL1 may exert similar protective effects on mitochondrial integrity, potentially through the modulation of inflammatory pathways that compromise mitochondrial homeostasis. Furthermore, other antioxidant-based strategies, including the use of SOD mimetics and ferroptosis inhibitors, have shown potential in AKI models. However, their clinical translation remains limited, often due to off-target effects or insufficient delivery to renal mitochondria [35]. In contrast, the advantage of targeting CX3CL1 lies in its upstream regulatory role, which may allow for broader protection by simultaneously suppressing inflammation and mitigating oxidative stress.

CX3CR1 is the only receptor proven to interact with CX3CL1. The CX3CR1 polypeptide chain binds to the CX3CL1 ligand extracellularly and forms a heterotrimeric G protein-coupling site by binding to the terminal polypeptide chain located on the cytoplasmic side. It has chemotactic functions and facilitates the adhesion of CX3CR1^+^ cells [22]. The expression of CX3CR1 is significantly increased in a mouse model of acute kidney injury, particularly in macrophages and monocytes in the kidneys. Research suggests that activation of the CX3CR1 receptor may promote the migration of these immune cells to the site of injury, thereby exacerbating local inflammatory responses [36]. Another study also found that inhibiting the CX3CL1/CX3CR1 axis improved the antioxidant status of rats with sepsis and suppressed inflammatory responses [12]. In our study, AZD8797 (a selective CX3CR1 antagonist) was used to intervene in LPS-mediated RAW264.7 macrophages. We found that inhibiting CX3CR1 could convert LPS-induced M1-type macrophages to M2-type and alleviate LPS-induced macrophage mitochondrial dysfunction. Our study confirmed the therapeutic potential of targeting the CX3CL1/CX3CR1 axis for acute kidney injury.

The upregulation of TFAM observed in the CX3CL1-deficient AKI model underscores its essential role in maintaining mtDNA integrity. Beyond its classical functions in mtDNA transcription and replication, TFAM also plays a structural role by packaging mtDNA into a nucleus-like configuration that shields it from oxidative damage and enzymatic degradation [20, 37]. In this study, TFAM expression was found to be reduced in the kidneys of AKI mice. Previous research has shown that TFAM knockdown alone is sufficient to induce mitochondrial ROS (mtROS) production, disrupt mtDNA nucleoid structure, and impair mitochondrial respiration in kidney tubular epithelial cells [38]. Our findings suggest that CX3CL1 signaling suppresses TFAM expression under inflammatory conditions, thereby rendering mtDNA more vulnerable to LPS-induced damage. In contrast, the inhibition of CX3CL1 led to increased TFAM expression and reduced mtDNA leakage in both the AKI model and in LPS-stimulated macrophages. Moreover, the protective effects of CX3CL1 inhibition on mtDNA structural integrity and mitochondrial respiration were abolished upon TFAM silencing, further supporting a mechanistic link between CX3CL1 signaling and TFAM regulation. While the precise molecular mechanism by which CX3CL1 regulates TFAM stability warrants further investigation. One possible mechanism is that CX3CL1-induced ROS could oxidize TFAM, rendering it prone to degradation. TFAM contains redox-sensitive cysteine residues critical for its DNA-binding activity [39], and mitochondrial ROS are known to disrupt TFAM-mtDNA nucleoid integrity [38]. The antioxidant effects of CX3CL1 inhibition (via ROS regulation) may thus preserve TFAM function. In addition, CX3CL1 may indirectly inhibit TFAM transcription by activating NF-κB. NF-κB is a known downstream effector of CX3CL1 signaling [40] and has been reported to repress PGC-1α in inflammatory conditions [41]. The inflammatory cytokines can downregulate TFAM through an NF-κB-dependent pathway, establishing a feedforward cycle of mitochondrial dysfunction and inflammation [42]. Another possible mechanism is the ubiquitin-proteasome degradation mechanism. Studies have shown that inflammatory stimuli (such as LPS) can accelerate TFAM degradation through the E3 ubiquitin ligase (MARCH5), thereby promoting mitochondrial dysfunction [43]. Previous studies have reported that PGC-1α acts as a transcriptional coactivator of nuclear factors NRF1/2, thereby promoting TFAM transcription [44]. Intriguingly, in our earlier work, we observed that CX3CL1 modulates the protein abundance of PGC-1α [16]. These findings suggest that the regulatory effect of CX3CL1 on TFAM expression may, at least in part, be mediated through PGC-1α, positioning CX3CL1 as an upstream modulator of the PGC-1α–NRF1/2–TFAM axis. Together, these data suggest that CX3CL1 plays a broader role in regulating mitochondrial stability and mtDNA maintenance in AKI macrophages than previously recognized.

The preservation of mtDNA integrity in CX3CL1-deficient AKI models carries important implications. mtDNA damage not only disrupts oxidative phosphorylation but, when released into the cytosol, also acts as a danger-associated molecular pattern (DAMP) that amplifies inflammation through activation of the cGAS-STING signaling pathway [45]. Our results demonstrate that CX3CL1 deficiency reduces Bax activation, indicative of decreased mitochondrial outer membrane permeabilization, as well as suppresses cGAS-STING pathway activity, suggesting that stabilization of mtDNA underlies the observed protection against mitochondrial dysfunction and inflammation. The coordinated regulation of Bax and cGAS-STING signaling observed in our model points to an integrated relationship between mitochondrial apoptosis and innate immune activation. Specifically, Bax-mediated outer membrane permeabilization facilitates mtDNA release into the cytosol, where it serves as a ligand for cGAS-STING signaling [46]. CX3CL1 appears to regulate this entire axis, as its deficiency not only preserves mitochondrial integrity by limiting Bax activation but also reduces cytosolic DNA sensing by inhibiting cGAS-STING signaling. This dual action may explain the pronounced protective effect of CX3CL1 deletion observed in our AKI models.

In the present study, we mainly focused on the role of macrophages and demonstrated that CX3CL1 deficiency promoted macrophage polarization from the pro-inflammatory M1 phenotype toward the anti-inflammatory M2 phenotype, thereby reducing inflammatory injury in AKI. However, we recognize that macrophages are not the only contributors to renal protection. The data shown in our studies also clearly indicate improved morphology and function of tubular epithelial cells (TECs) in CX3CL1 KO + LPS mice. Considering the central role of TECs in the initiation and progression of AKI, their contribution to the ameliorated phenotype cannot be ignored. We propose that the improvement of TECs may occur, at least in part, as a downstream effect of macrophage polarization. M1 macrophages secrete abundant pro-inflammatory cytokines that aggravate TEC injury, whereas M2 macrophages promote repair by releasing anti-inflammatory and pro-regenerative factors. Thus, the shift of macrophages toward an M2 phenotype in CX3CL1-deficient mice likely creates a favorable microenvironment that alleviates TEC injury. Therefore, a reciprocal interaction between macrophages and TECs is likely involved in mediating the protective effect of CX3CL1 deficiency in AKI. While macrophage polarization appears to be a major regulatory mechanism, the concomitant improvement of TECs may further contribute to the overall protective phenotype. Future studies focusing on macrophage –TECs crosstalk will be of great value in fully elucidating the underlying mechanisms.

Nevertheless, this study has several limitations. First, the relatively small sample size and lack of clinical specimen validation may limit the robustness and generalizability of the findings. Second, the use of primarily murine models raises questions about the translational relevance of these results to human AKI. Third, although this study focused on CX3CL1, other regulatory factors involved in AKI pathophysiology may contribute to macrophage polarization and mitochondrial instability and were not addressed here. Fourth, although our study identified CX3CL1 as an upstream regulator of TFAM stability, further experiments are needed to reveal its specific regulatory mechanism. Future research should aim to expand the scope of investigation to include additional pathways and validate findings in human tissues to support the development of targeted therapies.

**Fig. 11:**
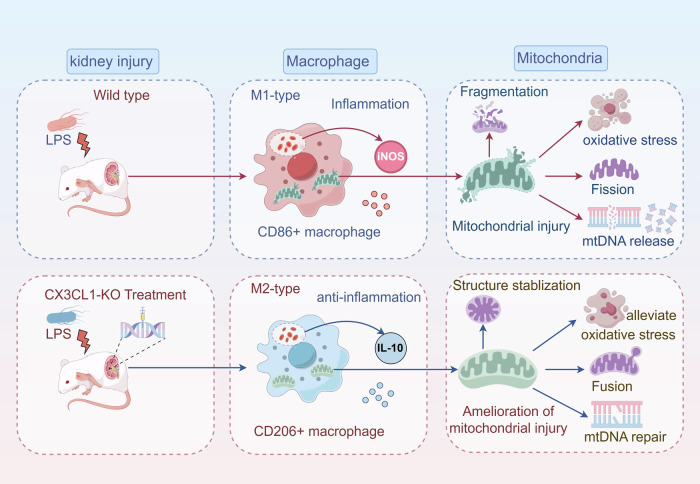
Schematic summary of the proposed mechanism. CX3CL1 deficiency alleviates macrophage mitochondrial dysfunction and promotes the phenotypic conversion from M1- to M2-type macrophages, thereby ameliorating kidney injury in LPS-induced AKI.

In conclusion, our results demonstrate that CX3CL1 plays a critical role in the pathogenesis of AKI, particularly by influencing macrophage polarization and mitochondrial homeostasis. CX3CL1 deficiency significantly improved kidney function in AKI mice, reduced inflammation, promoted a phenotypic shift of macrophages from M1 to M2 type, and stabilized mitochondrial structure and mtDNA integrity (Fig. 11). These findings highlight the therapeutic potential of CX3CL1 inhibition and underscore the multifaceted role of CX3CL1 in modulating immune responses and mitochondrial dynamics. Overall, this study provides important insights into the immunometabolic regulation of AKI and presents CX3CL1 as a promising target for future clinical intervention.

## Supplementary information

supplementary file

Sequence data

## Data Availability

The original data supporting the findings of this study are presented within the article’s Materials section. Further inquiries can be sent to the corresponding authors.
