# Supplementary material for: CX3CL1 deficiency ameliorates acute kidney injury by inhibiting macrophage mitochondrial dysfunction and mtDNA-cGAS-STING signaling
Source: Cell Death Discov. 2025 Dec 13;12:69. doi: 10.1038/s41420-025-02915-w (PMC12847948; doi:10.1038/s41420-025-02915-w)

Table S1 The primer sequences

Gene	Primer sequence
Mouse GAPDH	F:5'- AGGTCGGTGAACGGATTG-3'<br>R:5'- TGTAGACCATGTAGTTGAGGTCA-3'
Mouse IL-1β	F:5'-CAACCAACAAGTGATATTCTCCATG-3'<br>R:5'-GATCCACACTCTCCAGCTGCA-3'
Mouse IL-6	F:5'- ACAACCACGGCCTTCCCTACTT-3'<br>R: 5'- CACGATTTCCCAGAGAACATGTG-3'
Mouse MCP-1	F:5'- TTAAAAACCTGGATCGGAACCAA-3'<br>R:5'- GCATTAGCTTCAGATTTACGGGT-3'
Mouse ICAM-1	F:5'- ACCCAACTGGAAGCTGTTTG -3'<br>R:5'- CACACTCTCCGGAAACGAAT -3'
Mouse TFAM	F:5'- CACCCAGATGCAAAACTTTCAG -3'<br>R:5'- CTGCTCTTTATACTTGCTCACAG -3'

Western blot

Figure. 1

TNF-α

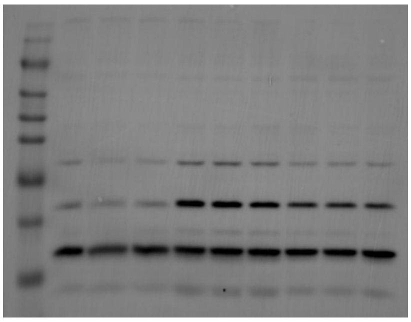

CX3CL1

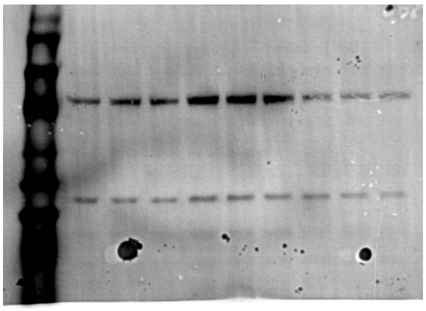

NGAL

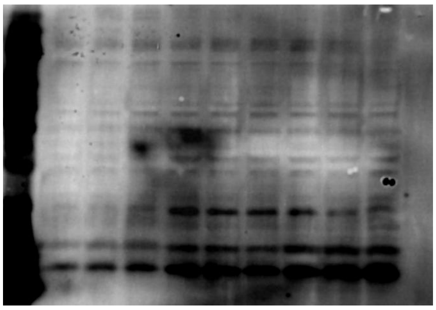

Kim-1

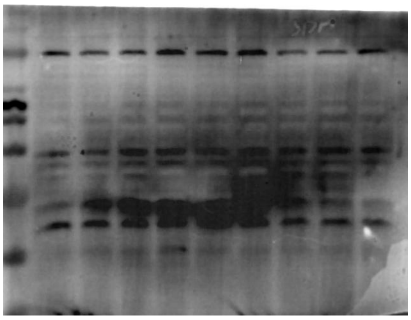

β-actin

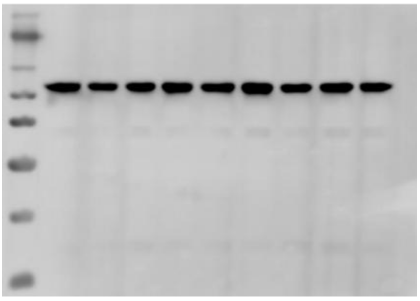

Figure.3 A

CD86

iNOS

IL-10

CD206

β-actin

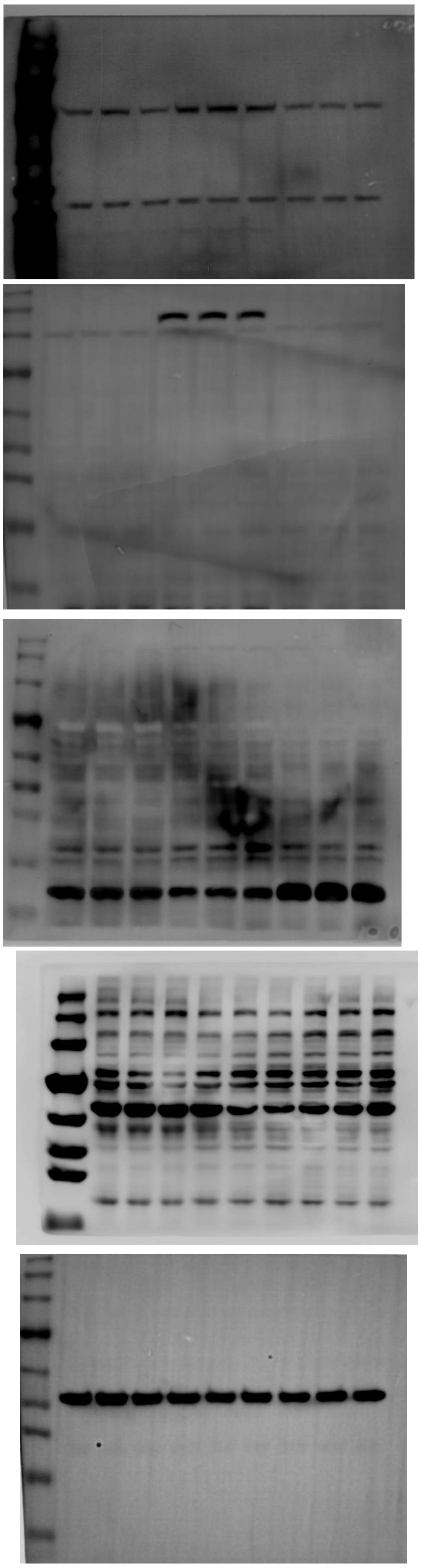

Figure.3 F

CD86

iNOS

IL-10

CD206

β-actin

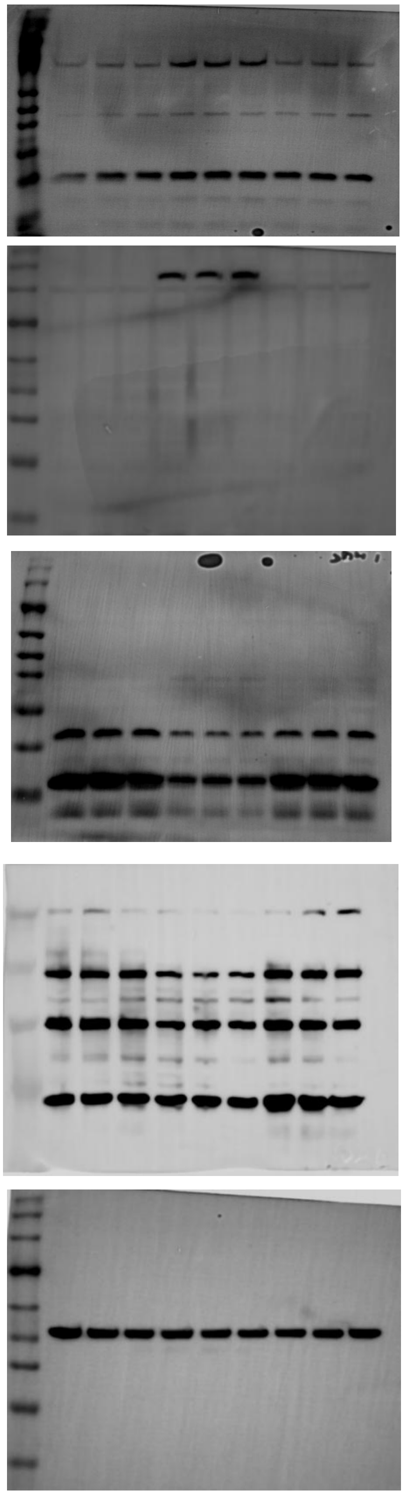

Figure.3 I

CX3CL1

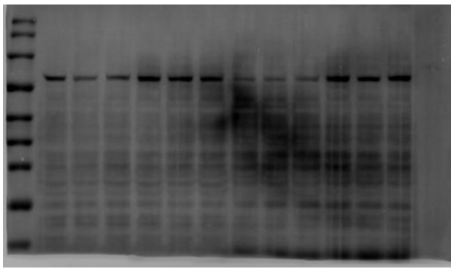

CD86

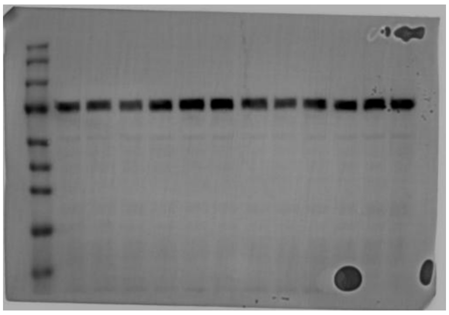

iNOS

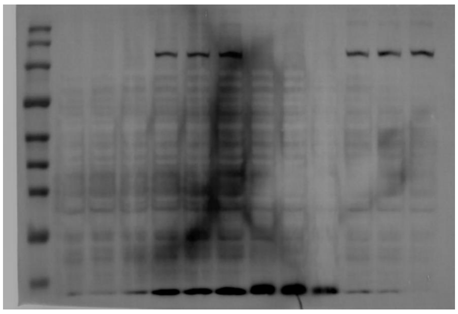

IL-10

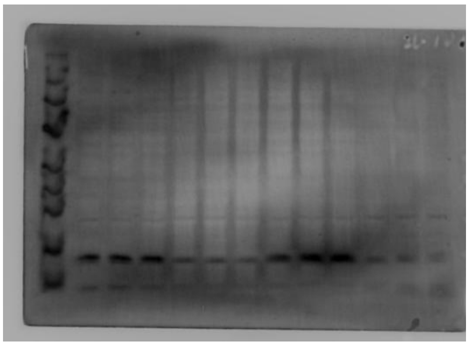

CD206

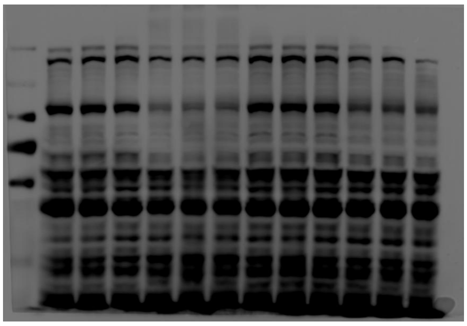

β-actin

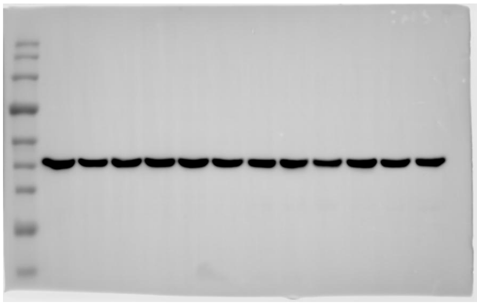

Figure.4D

ATPB

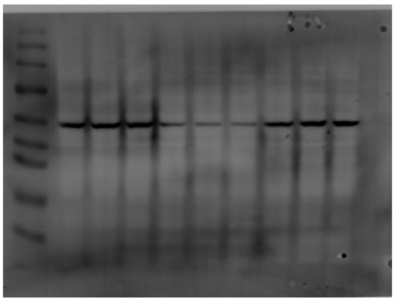

Tomm20

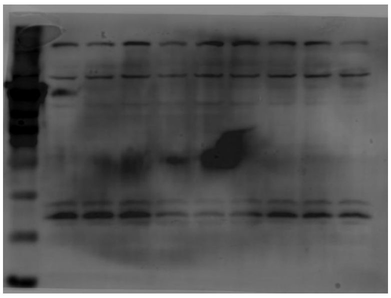

SOD2

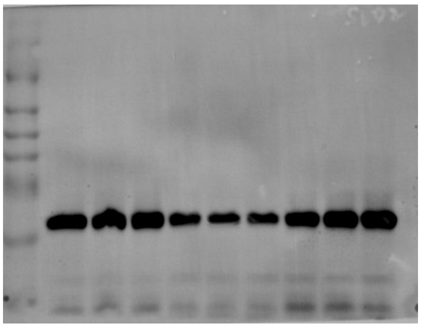

β-actin

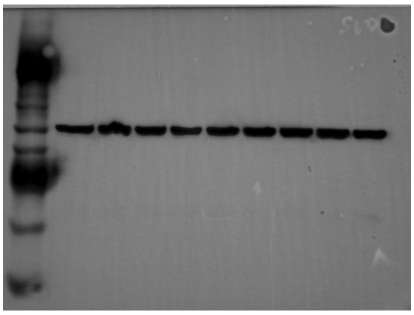

Figure.4J

ATPB

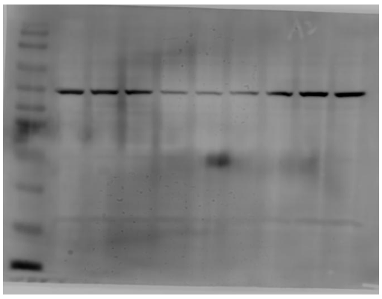

Tomm20

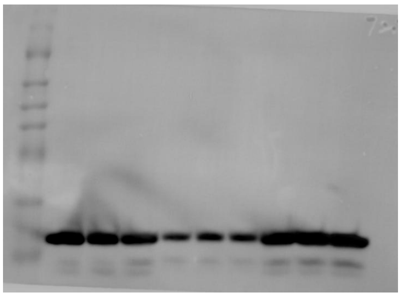

SOD2

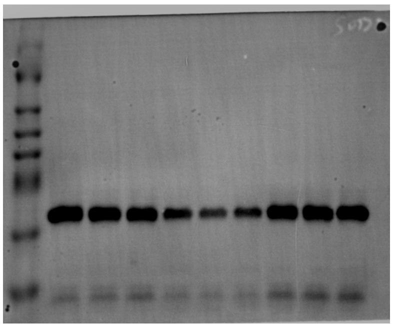

β-actin

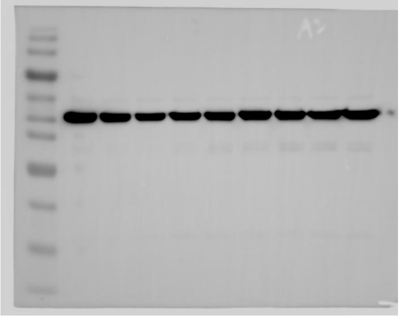

Figure.4 K

SOD2

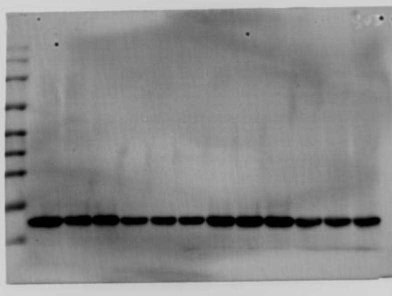

β-actin

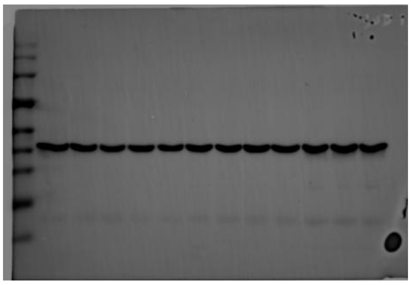

Figure.5A

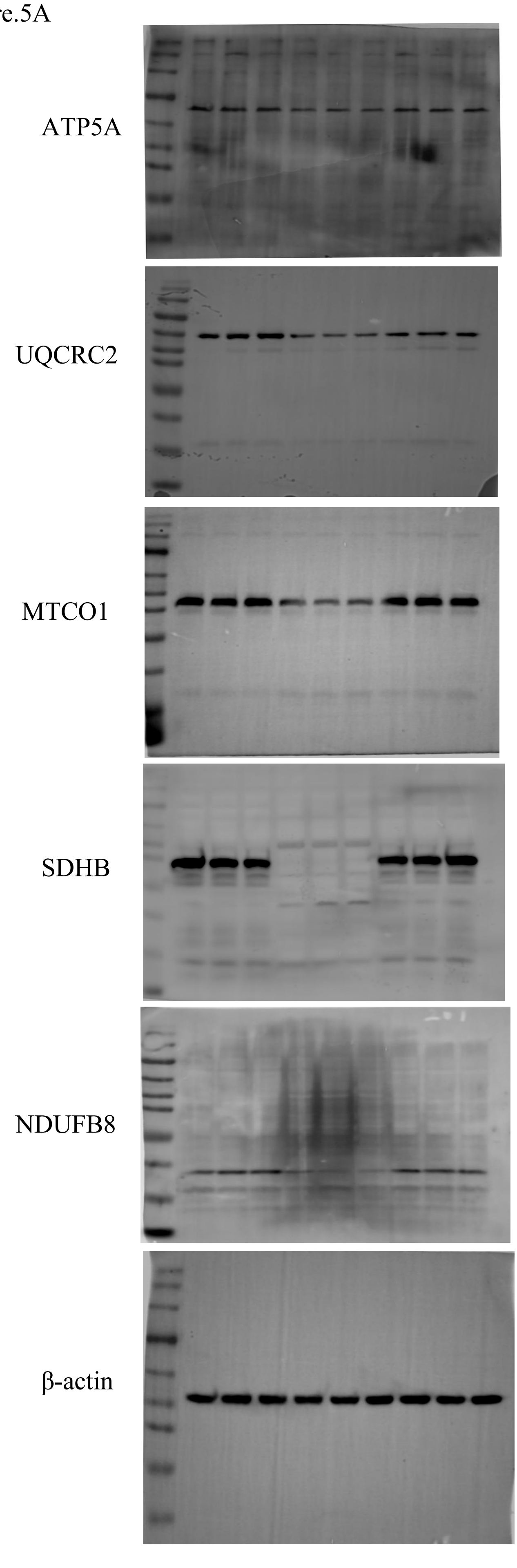

Figure.6B

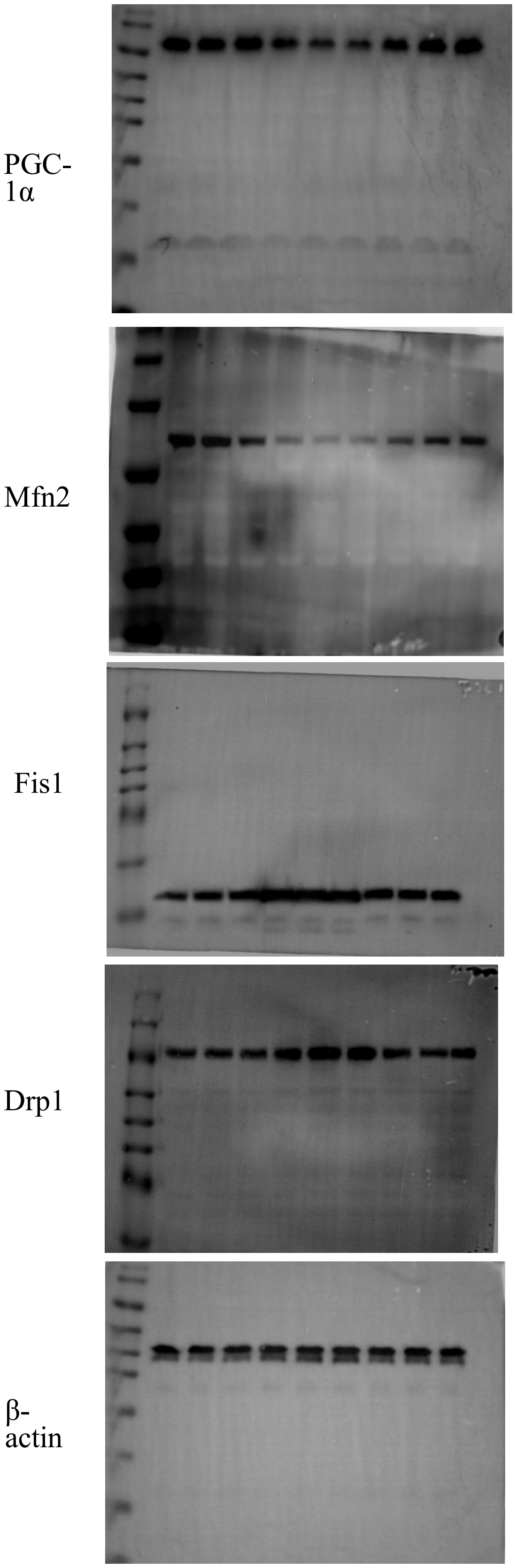

Figure.6E

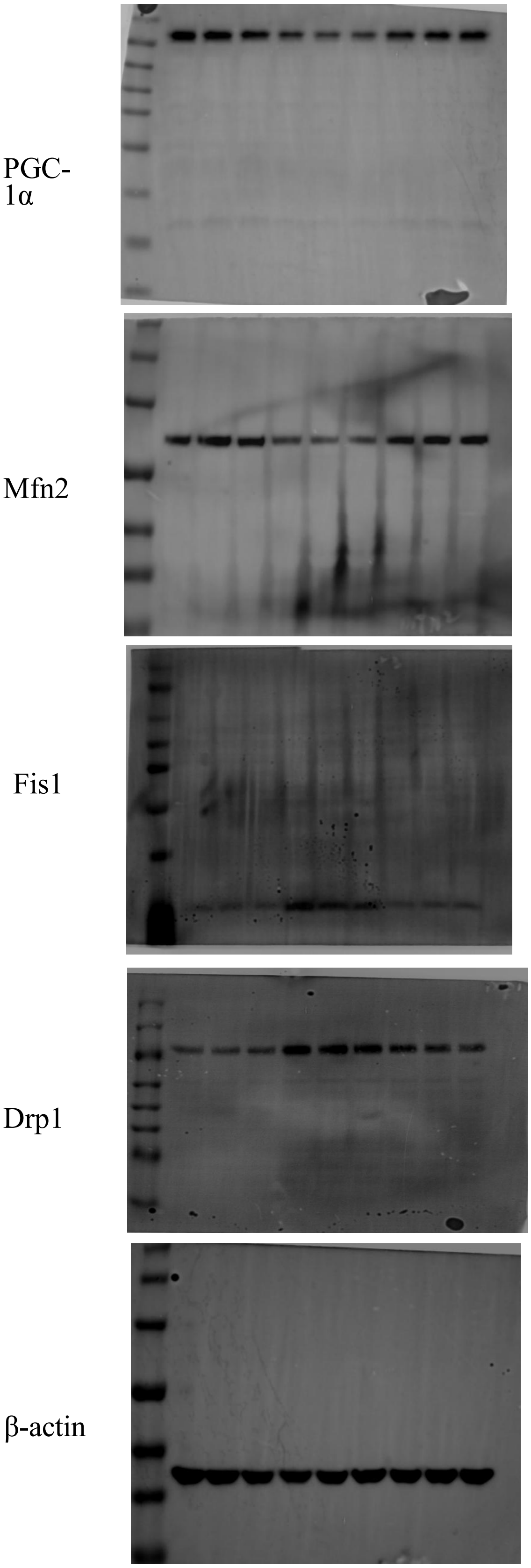

Figure.6G

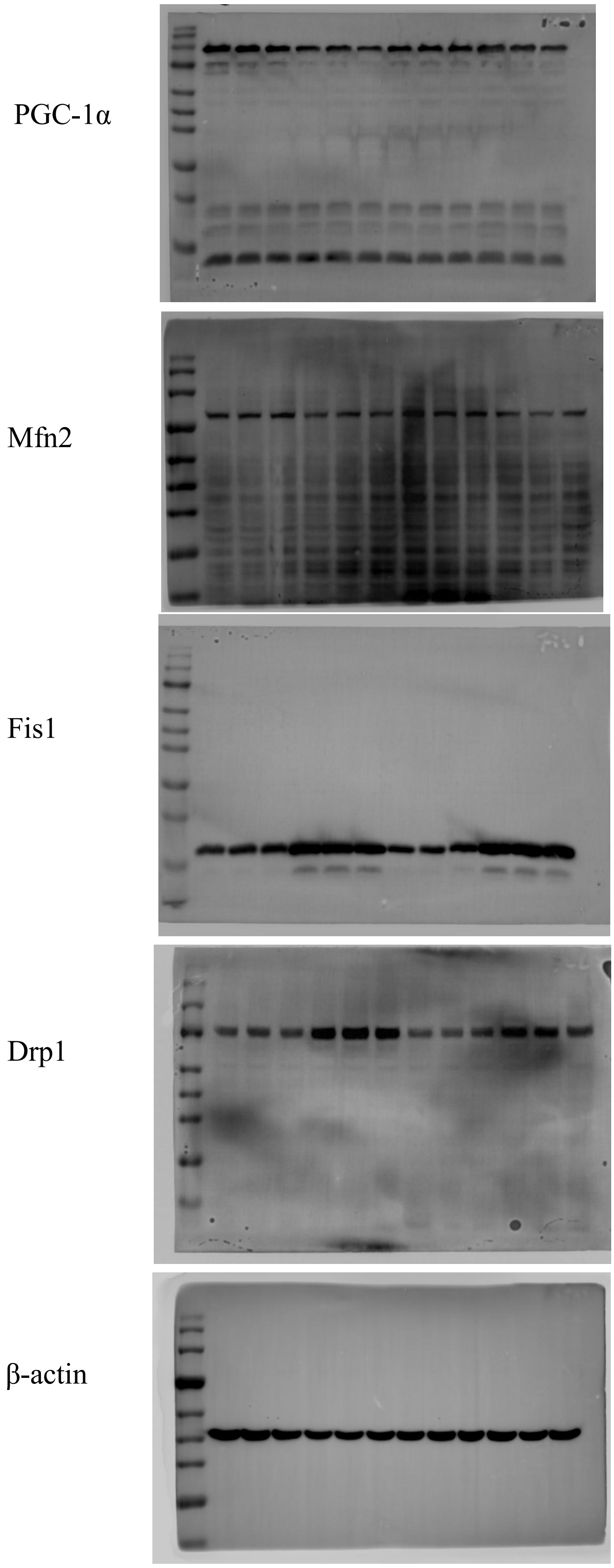

Figure.7A

TFAM

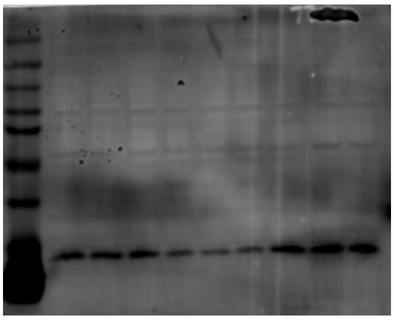

β-actin

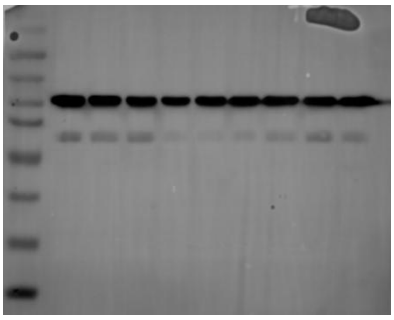

Figure.7D

TFAM

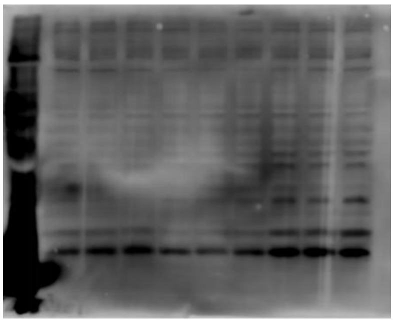

β-actin

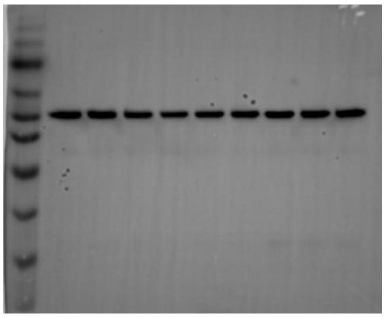

Figure.7G

TFAM

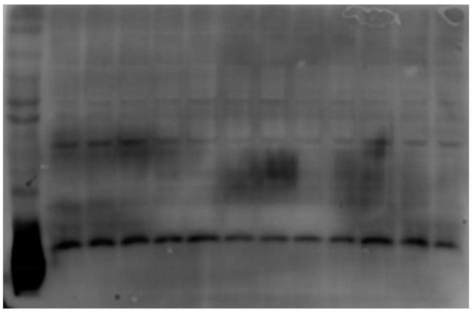

β-actin

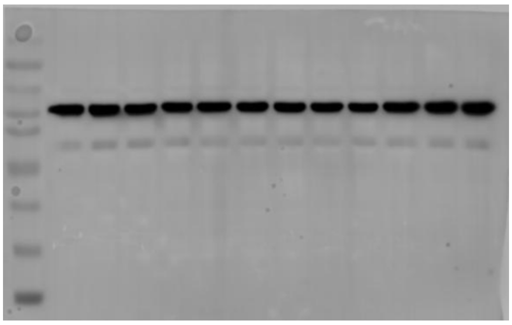

Figure.8A

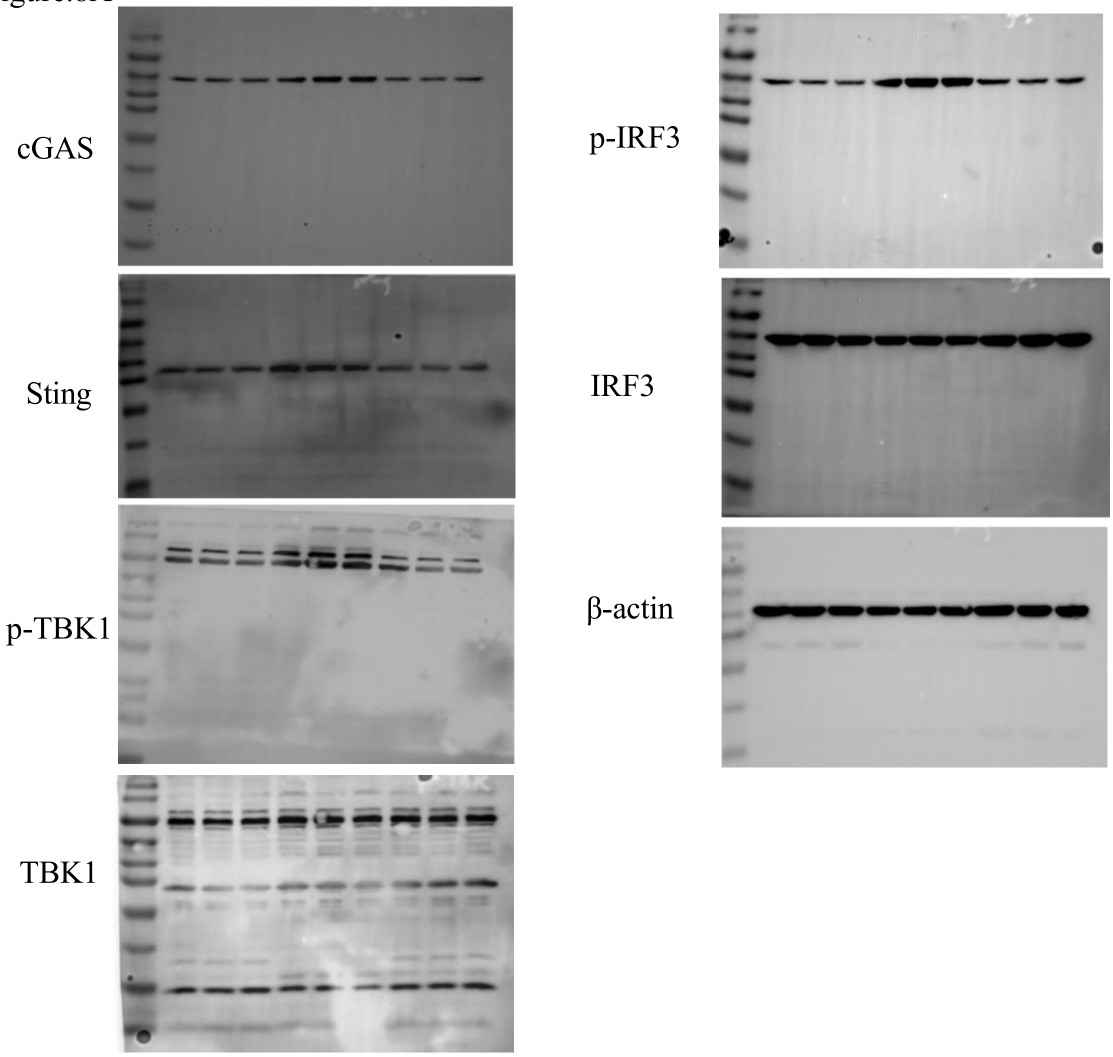

Figure.8B

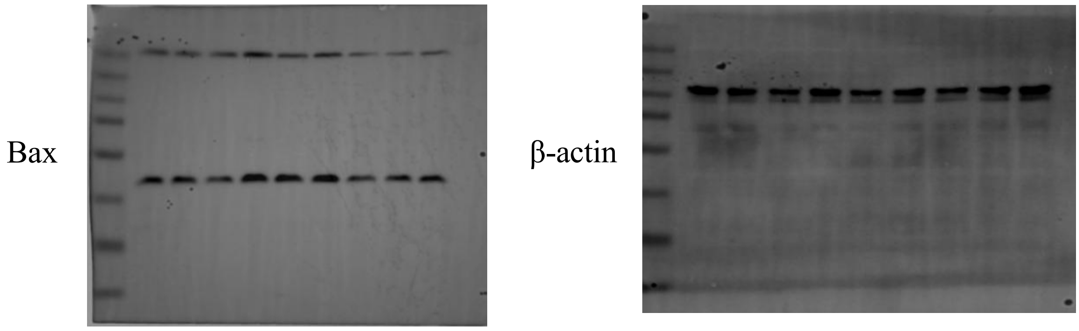

Figure.8D

cGAS

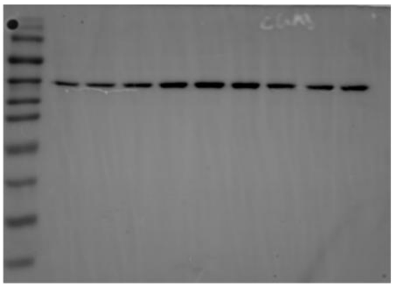

p-IRF3

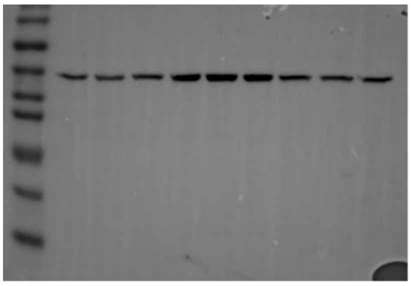

Sting

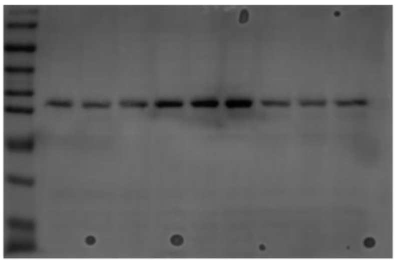

IRF3

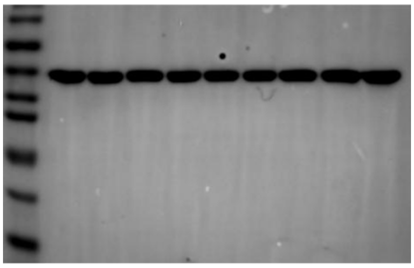

p-TBK1

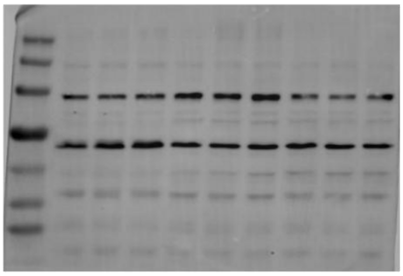

β-actin

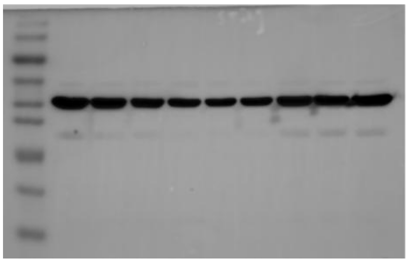

TBK1

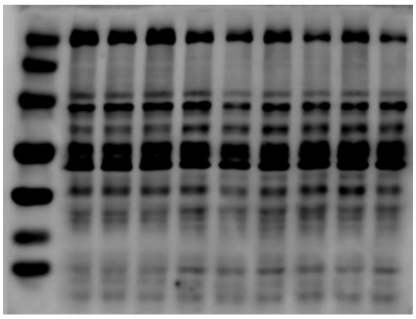

Figure.8E

Bax

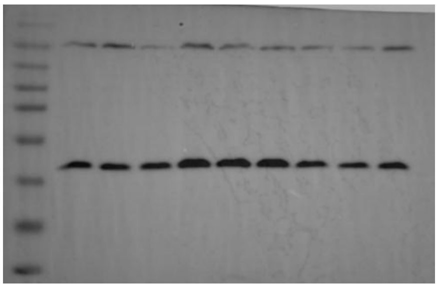

β-actin

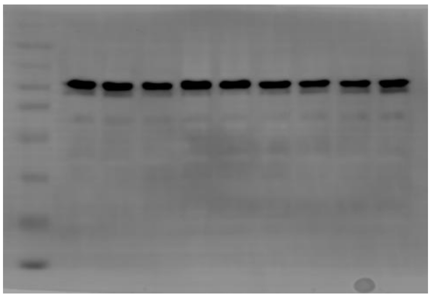

Figure.8G

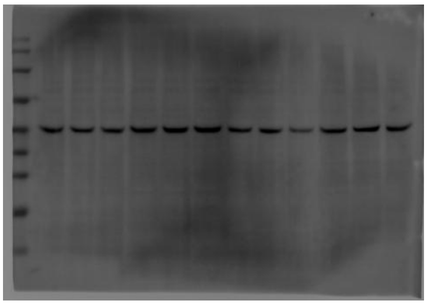

cGAS

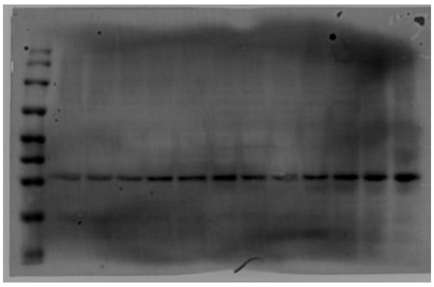

Sting

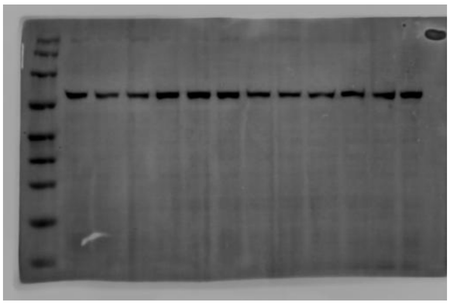

p-TBK1

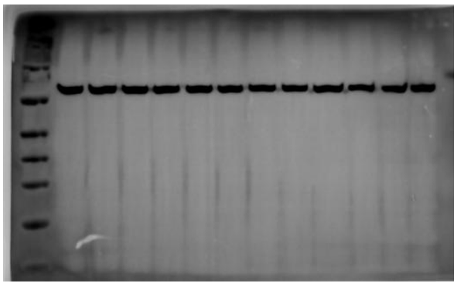

TBK1

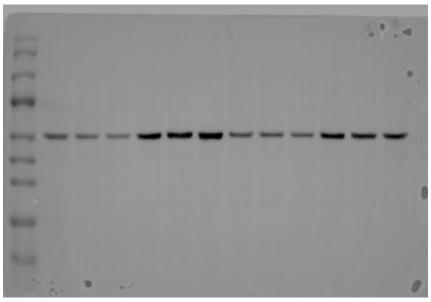

P-
IRF3

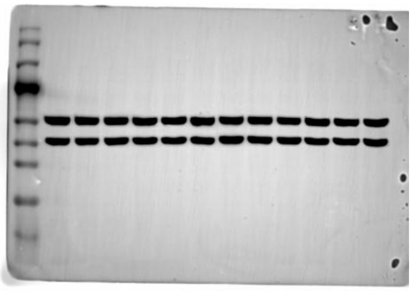

IRF3

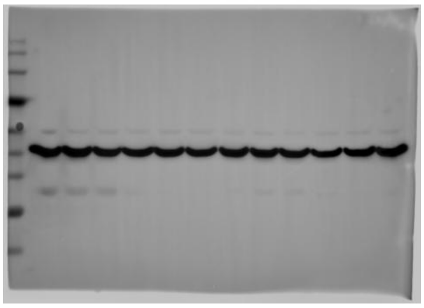

β-
actin

Figure.9A

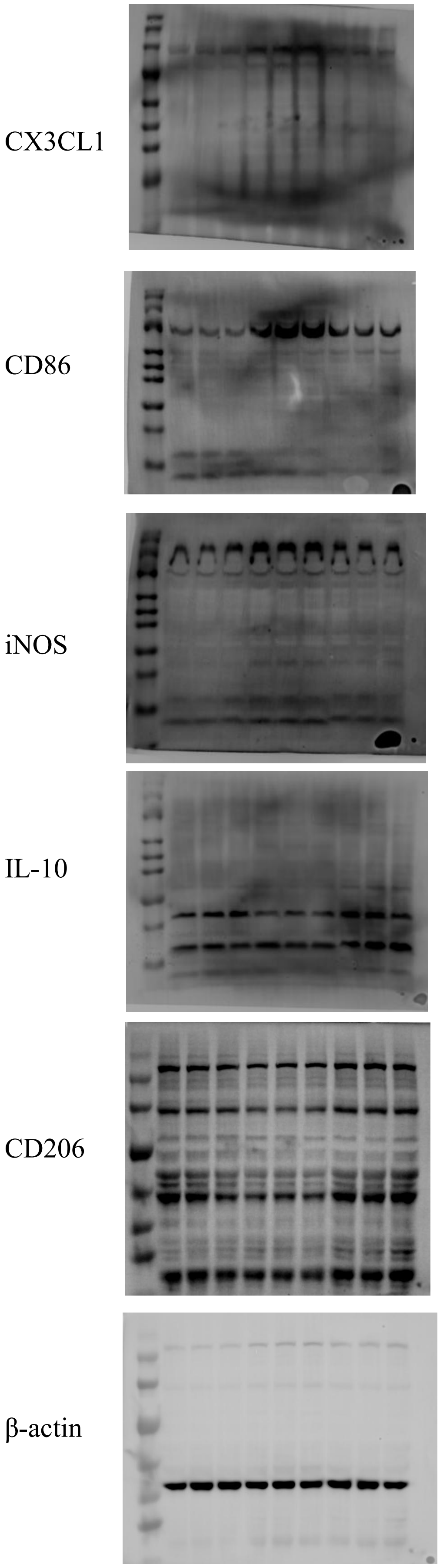

Figure.9C

PGC-1α

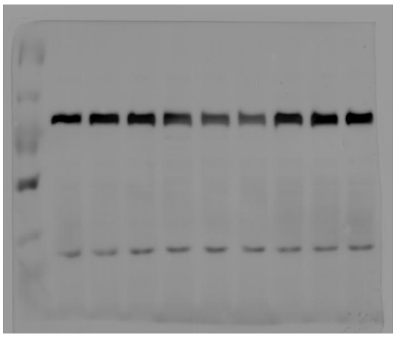

Mfn2

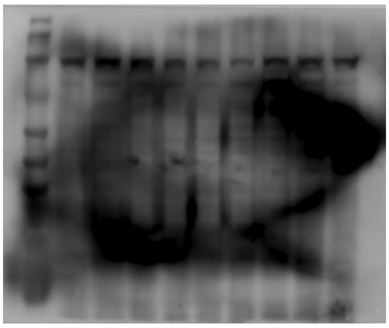

Fis1

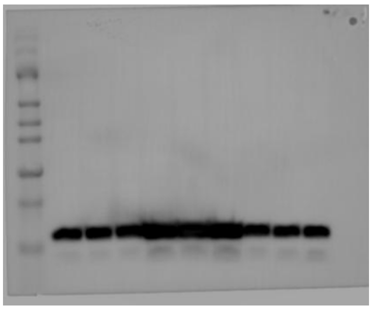

Drp1

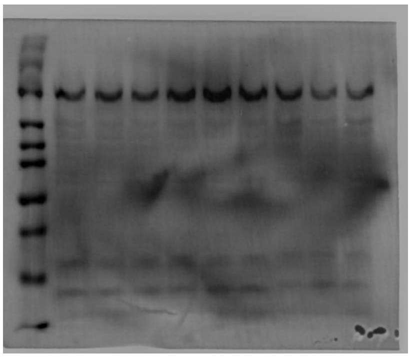

SOD2

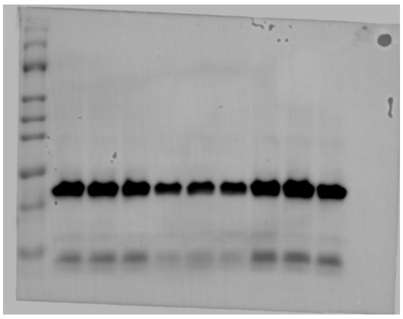

β-actin

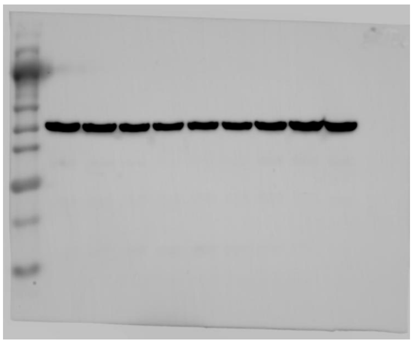

Figure.10A

Cx3cl1

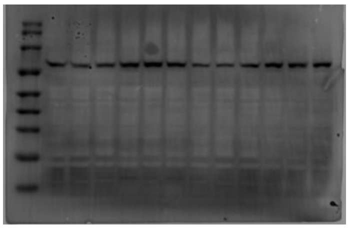

CD86

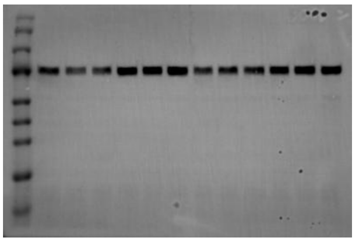

iNOS

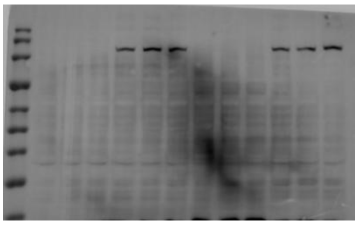

IL-10

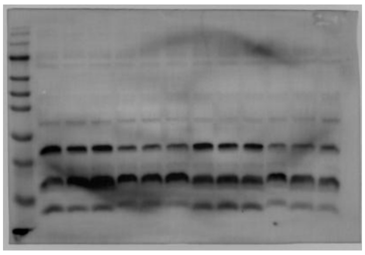

CD206

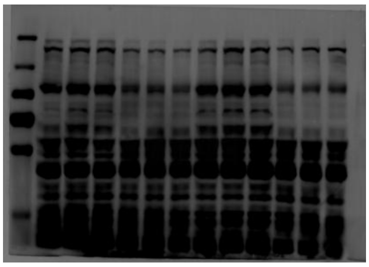

β-actin

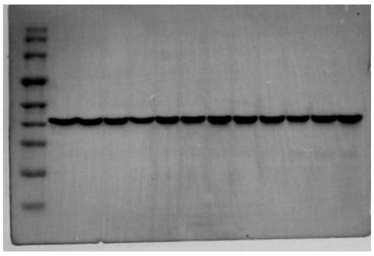

Figure.10C

PGC-1α

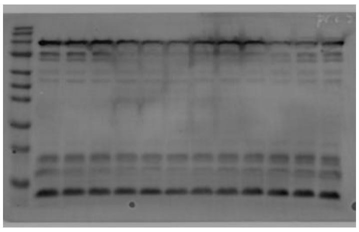

Mfn2

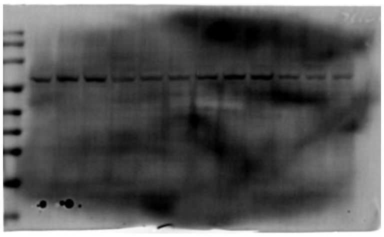

Fis1

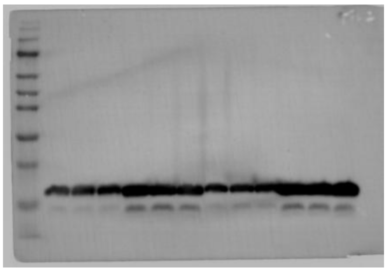

Drp1

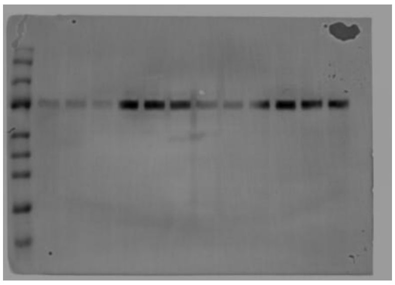

SOD2

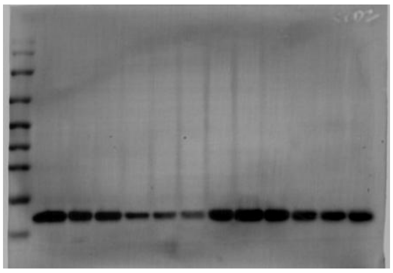

β-actin

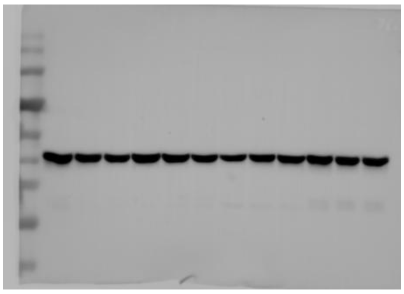

Figure.10F

cGAS

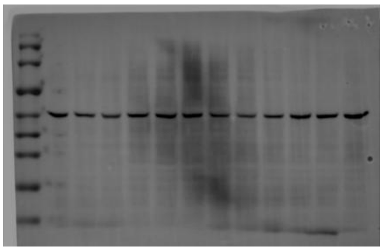

p-IRF3

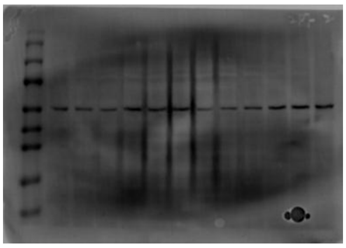

Sting

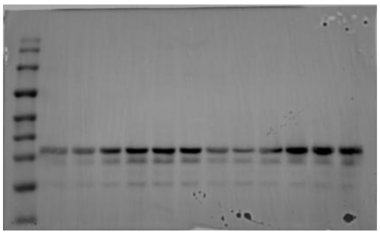

IRF3

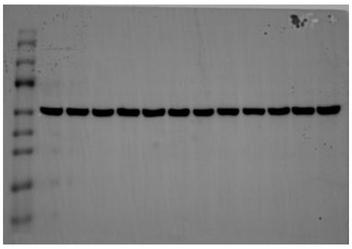

p-TBK1

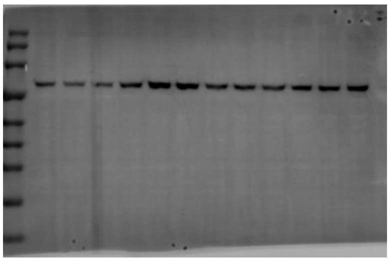

β-actin

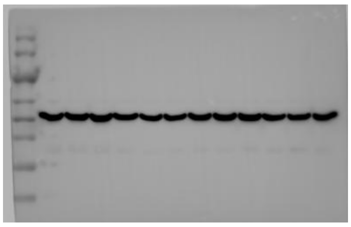

TBK1

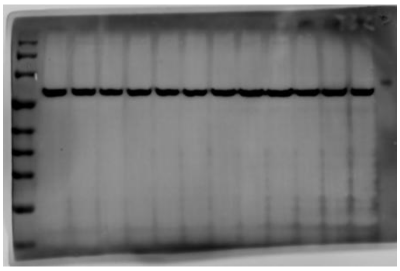

Figure.10E

TFAM

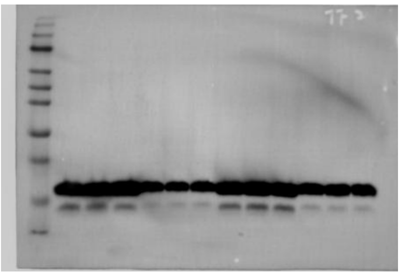

β-actin

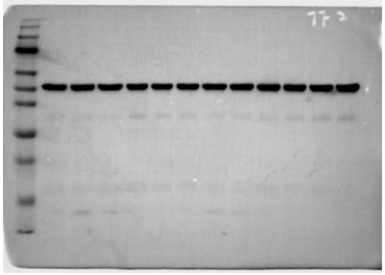

Supplement: Supplementary file 1 — supplementary file [file 41420_2025_2915_MOESM1_ESM.pdf]
